# Association of Diet and Waist-to-Hip Ratio With Brain Connectivity and Memory in Aging

**DOI:** 10.1001/jamanetworkopen.2025.0171

**Published:** 2025-03-12

**Authors:** Daria E. A. Jensen, Klaus P. Ebmeier, Tasnime Akbaraly, Michelle G. Jansen, Archana Singh-Manoux, Mika Kivimäki, Enikő Zsoldos, Miriam C. Klein-Flügge, Sana Suri

**Affiliations:** 1 Department of Psychiatry, University of Oxford, Oxford, United Kingdom; 2 Oxford Centre for Human Brain Activity, Wellcome Centre for Integrative Neuroimaging, University of Oxford, Oxford, United Kingdom; 3 Department of Neurology, Max Planck Institute for Human Cognitive and Brain Sciences, Leipzig, Germany; 4 Clinic for Cognitive Neurology, University of Leipzig Medical Center, Leipzig, Germany; 5 UCL Brain Sciences, University College London, London, United Kingdom; 6 Desbrest Institute of Epidemiology and Public Health, Université de Montpellier, Institut National de la Santé et de la Recherche Médicale (INSERM), Montpellier, France; 7 Donders Centre for Cognition, Donders Institute for Brain, Cognition and Behaviour, Radboud University, Nijmegen, the Netherlands; 8 Department of Neurology, Donders Institute for Brain, Cognition and Behavior, Radboud University Medical Center, Nijmegen, the Netherlands; 9 Epidemiology of Ageing and Neurodegenerative Diseases, Université Paris Cité, INSERM U1153, Paris, France; 10 Department of Experimental Psychology, Wellcome Centre for Integrative Neuroimaging, University of Oxford, Oxford, United Kingdom

## Abstract

**Importance:**

Epidemiological studies suggest that lifestyle factors are associated with risk of dementia. However, few studies have examined the association of diet and waist to hip ratio (WHR) with hippocampus connectivity and cognitive health.

**Objective:**

To ascertain how longitudinal changes in diet quality and WHR during midlife are associated with hippocampal connectivity and cognitive function in later life.

**Design, Setting, and Participants:**

This cohort study analyzed data from participants in the Whitehall II Study at University College London (study inception: 1985) and Whitehall II Imaging Substudy at the University of Oxford (data collection: 2012-2016). Healthy participants from the Whitehall II Imaging Study with a mean age of 48 years at baseline to 70 years at magnetic resonance imaging (MRI) were included if they had information on diet from at least 1 wave, information on WHR from at least 2 waves, and good-quality MRI scans. Study analyses were completed from October 2019 to November 2024.

**Exposures:**

Diet quality was measured in participants(mean age, 48 years at baseline to 60 years) using the Alternative Healthy Eating Index–2010 score, which was assessed 3 times across 11 years. WHR was measured 5 times over 21 years in participants aged 48 to 68 years.

**Main Outcomes and Measures:**

White matter structural connectivity assessed using diffusion tensor imaging, hippocampal functional connectivity assessed using resting-state functional MRI, and cognitive performance measures. Brain imaging and cognitive tests were performed at a mean (SD) age of 70 (5) years.

**Results:**

The final diet quality sample comprised 512 participants (403 males [78.7%]; mean [SD] age, 47.8 [5.2] years), and the final WHR sample included 664 participants (532 males [80.1%]; mean [SD] age, 47.7 [5.1] years). Better diet quality in midlife and from midlife to late life was associated with higher hippocampal functional connectivity to the occipital lobe and cerebellum (left hippocampus: 9176 mm^3^, *P* < .05; left hippocampus and to the right cerebellum: 136 mm^3^, *P* = .04) and better white matter integrity as measured by higher fractional anisotropy (FA; 19 432 mm^3^, *P* < .05) and lower diffusivity (mean diffusivity [MD]: 5560 mm^3^, *P* < .05; axial diffusivity [AD]: 2600 mm^3^, *P* < .045; AD in fornix: β [SE] = 0.26 [0.11], false discovery rate–corrected *P* = .02). Higher WHR in midlife was associated with higher MD and radial diffusivity (covering 26.4% [333 088 mm^3^, *P* < .001] and 23.1% [291 888 mm^3^, *P* < .05], respectively, of the total white matter tracts in the cingulum and superior and inferior longitudinal fasciculus) and lower FA in the corticospinal tract (covering 4.9% of the white matter skeleton), including the inferior longitudinal fasciculus and cingulum (61 272 mm^3^, *P* < .05). Associations between midlife WHR, working memory, and executive function were partially mediated by diffusivity (eg, digit span was mediated by global FA: β = –2.96^−03^; 95% CI, –5.56^−03^ to −1.01^−03^; *P* < .001).

**Conclusions and Relevance:**

This cohort study found that healthier diets and lower WHR throughout midlife were associated with better brain and cognitive health in older age. The findings suggest that interventions to improve diet and manage central obesity might be most effective between ages 48 and 70 years.

## Key Points

**Question:**

Are diet quality and abdominal fat, measured by waist to hip ratio (WHR), associated with brain connectivity and cognitive decline?

**Findings:**

In this cohort study of 512 participants in the diet quality cohort and 664 in the WHR cohort, better diet quality in midlife and across middle to older age was associated with enhanced hippocampal functional connectivity and white matter integrity. In contrast, higher WHR in midlife was associated with poorer working memory and executive function, through a pathway partially mediated by alterations in white matter connectivity.

**Meaning:**

These findings suggest that interventions to improve diet and manage central obesity might be best targeted in middle to older age.

## Teaser

This cohort study examines the association between dietary intake and waist to hip ratio in middle age and brain connectivity and cognitive decline in later years.

## Introduction

The global shift toward unhealthy dietary habits is associated with an increase in the prevalence of diabetes, cardiovascular disease,^1^ and obesity,^2^ all of which are known risk factors for dementia.^3^ The World Health Organization guidelines recommend a balanced diet with a high plant intake (eg, the Mediterranean diet) and weight management to reduce the risk of dementia.^4^ It is, therefore, important to consider the implications of overall diet and central obesity for memory and associated brain regions, such as the hippocampus.

A systematic review^5^ highlighted the need to understand the association between long-term adherence to healthy diets and brain connectivity and memory. Most studies on hippocampal and white matter connectivity^6,7^ have examined single dietary components, such as beetroot juice, omega-3 and omega-6 fatty acids,^5^ resveratrol,^8^ and caloric restriction,^9^ although the cognitive impacts have remained unclear. Given the importance of the hippocampus in Alzheimer disease, studies have also investigated the extent to which both obesity and poor diet quality might alter hippocampal volume, but the evidence on the associations with hippocampal connectivity is inconsistent.^5^ Lower hippocampal activity has been linked to worse memory of meals (ie, forgetting when the last meal was consumed), reduced meal intervals, and increased response to food cues,^10^ which play a role in lower dietary quality and higher body mass index (BMI). This region merits further consideration in the context of the diet-brain links.^11^ Moreover, the timing and direction of the diet-brain associations are poorly characterized due to a lack of longitudinal studies, particularly during the transition period from middle to older age (ie, 40 to 70 years). This period has been identified as a key window for preventive interventions to reduce dementia risk.^3^

In this study, we aimed to ascertain the association of diet quality and waist to hip ratio (WHR) in midlife as well as their longitudinal changes during middle to older age with (1) structural and functional connectivity of the hippocampus and (2) cognitive function in later life. We used data from comprehensive dietary questionnaires and measurements of WHR over a 21-year period in midlife. We hypothesized that higher diet quality and lower WHR throughout midlife are associated with more favorable indicators of hippocampal functional and structural connectivity, which were measured using resting-state functional magnetic resonance imaging (fMRI) and diffusion tensor imaging (DTI). Our focus was on DTI markers of myelin and axonal integrity, such as fractional anisotropy (FA) and diffusivity. We further hypothesized that the associations of diet and WHR with cognitive function are mediated by these brain outcome measures.

## Methods

### Sample Selection

The Whitehall II Study, established in 1985 by University College London, is a longitudinal study of 10 308 individuals from the British Civil Service who have been followed up for over 30 years through 13 study waves.^12^ WHR was measured at waves 3 (1991-1994), 5 (1997-1999), 7 (2002-2004), 9 (2007-2009), and 11 (2012-2013), while diet was assessed at waves 3, 5, and 7. A random subset of participants also received brain MRI scans and cognitive tests as part of the Whitehall II Imaging Substudy, which was conducted at the Wellcome Centre for Integrative Neuroimaging, University of Oxford, from 2012 to 2016.^13^ The 3T MRI scans were obtained shortly after wave 11 for 775 participants according to the published protocol^13^ (eMethods 1 and eTable 1 in Supplement 1). Ethical approval was obtained in accordance with the Declaration of Helsinki.^14^ Written informed consent was obtained from all participants at each Whitehall II Study data collection. This prospective cohort study was approved by the University of Oxford Central University Research Ethics Committee and the University College London Medical School Committee on the Ethics of Human Research. We followed the Strengthening the Reporting of Observational Studies in Epidemiology (STROBE) reporting guideline.

We included participants of the Whitehall II Imaging Substudy who had information on diet from at least 1 of 3 previous waves; information on WHR from at least 2 of 5 previous waves; and good-quality structural, resting-state fMRI and DTI scans. The sample selection flowchart is provided in eMethods 2 and eFigure 1 in Supplement 1. Any missing diet and WHR data from individual waves were imputed for each participant using the nearest-neighbor interpolation. The final sample for the fMRI analysis consisted of 664 participants for analyses of WHR and 512 participants for analyses of diet quality. For the DTI analyses, 8 participants in the WHR group and 6 participants from the diet group were excluded because of missing or poor-quality DTI data, resulting in a final sample of 657 participants for WHR and 506 participants for diet groups.

### Alternative Healthy Eating Index–2010 Score

Dietary intake was assessed in waves 3, 5, and 7 of the Whitehall II Study using a validated machine-readable Food Frequency Questionnaire (FFQ), which included 127 food items commonly eaten in the UK.^15^ The FFQ data were used to assess diet quality with the Alternative Healthy Eating Index–2010 (AHEI-2010) score.^16,17^ Built on 11 components, the AHEI-2010 has a score range of 0 to 110, with higher scores representing a healthier diet. Scoring criteria for the AHEI-2010 and its distribution are described in eMethods 3 in Supplement 1.

### WHR

While most studies have used BMI (calculated as weight in kilograms divided by height in meters squared) as a proxy marker for obesity, more recent literature has highlighted WHR as a potentially more accurate factor in health and disease.^18^ In the present study, WHR was estimated by dividing the waist circumference at the biggest diameter by the trochanter circumference. We used WHR data that were obtained before brain scans, including waves 3, 5, 7, 9, and 11.

### Longitudinal Changes in Diet and WHR

We used linear mixed-effects models with maximum likelihood estimation to extract the intercept (projected diet at wave 3) and slope (change in diet over time from middle to older age) for diet and WHR; individual trajectories are shown in eFigure 2 in Supplement 1. Both the intercepts and slopes (time) were fitted as random effects. The best-fit model was the linear model for diet and a natural cubic spline model with 2 *df* for WHR (eMethods 4, eTables 2-4, and eFigure 3 in Supplement 1).

### Structural and Functional Connectivity

White matter microstructure was assessed using DTI scans analyzed with tract-based spatial statistics.^19^ We extracted global FA, radial diffusivity (RD), axial diffusivity (AD), and mean diffusivity (MD) from the mean tract-based spatial statistics skeleton as proxy markers of myelin integrity (eMethods 5 in Supplement 1). We additionally extracted FA, MD, RD, and AD values from 3 regions of interest (ROIs) in proximity to the hippocampus based on the literature^20^: the fornix, the inferior longitudinal fasciculus (ILF), and the cingulum.

Hippocampal functional connectivity was analyzed using seed-based correlation analyses with a hippocampus seed mask,^21^ which was smoothed using a kernel sphere of 4 mm radius (eMethods 5 and eFigure 4 in Supplement 1^22^) with 1000 permutations. Mean hippocampal connectivity values were extracted from significant clusters and plotted for visualization.

### Cognitive Tests

Cognitive tests were performed at the time of the MRI scan. We examined 3 cognitive domains: working memory (short-term memory recall of words on the Hopkins Verbal Learning Test–Revised and the total score on the Digit Span Forward, Backward, and Sequence Tests), fluency (semantic fluency: number of animals named in 1 minute; lexical fluency: number of words beginning with the letter F in 1 minute), and executive function (difference in time to completion between Trail Making Test parts A and B and the total score on the Digit Coding Test adapted from the Wechsler Adult Intelligence Scale–Fourth Edition). Overall cognitive health was ascertained using the Montreal Cognitive Assessment (score range: 0-30, with a score above 26 generally indicating normal cognitive performance; optimal cutoffs vary by population).

### Statistical Analysis 

Analyses were performed from October 2019 to November 2024. R, version 2.3.1 (R Project for Statistical Computing) and FSL, version 6.0.5 (Wellcome Centre for Integrative Neuroimaging) were used in analyses.

To examine the association between the intercepts and slopes of AHEI-2010 and WHR with (1) structural connectivity, (2) functional connectivity, and (3) cognitive outcomes, we performed linear regression. Based on evidence linking brain connectivity to diet,^5,6,7^ body weight or obesity,^23,24,25,26,27,28,29,30^ and cognition,^24,31,32,33^ we tested mediation models to examine whether the observed associations between AHEI-2010 or WHR and cognition were mediated by brain connectivity. We tested mediation only if there was a direct association of the intercepts and slopes of AHEI-2010 or WHR with cognitive outcomes (X→Y). In that case, we tested those brain connectivity mediators (M) that were associated with AHEI-2010 or WHR (X→M) (eMethods 6 in Supplement 1).

All analyses were adjusted for age, sex, years of education, MRI scanner model, physical activity, and the Montreal Cognitive Assessment score, all of which were measured at the time of MRI scan. Resting-state fMRI analyses were corrected for head motion and voxelwise gray matter density. AHEI-2010 analyses were adjusted for total energy intake (kcal/d) (eMethods 7 in Supplement 1).

To correct for multiple comparisons, we used the Benjamini-Hochberg false discovery rate (FDR) for the 6 intercorrelated cognitive tests and Bonferroni correction for the 3 white matter tracts. Statistical significance was set at *P* < .017 for the white matter ROI analysis across 3 predefined tracts. The group-level voxelwise analysis included strict threshold-free cluster enhancement and a correction for familywise errors (*P* < .05) for multiple voxelwise comparisons.

## Results

After exclusions, the final diet quality sample comprised 512 participants and the final WHR sample included 664 participants. The diet cohort included 403 males (78.7%) and 109 females (21.3%), with a mean (SD) age of 47.8 (5.2) years at wave 3 (baseline; 1991-1994), 59 (5.1) years at wave 7, and 69.8 (5.1) years at the time of MRI scan (2012-2016). The WHR cohort included 532 males (80.1%) and 132 females (19.9%), with a mean (SD) age of 47.7 (5.1) years at wave 3, 67.9 (5.1) years at wave 11, and 69.8 (5.1) years at MRI scan (eTable 5 in Supplement 1). Brain imaging and cognitive tests were performed at a mean (SD) age of 70 (5) years. The mean (SD) BMI for both groups was 26 (4).

**Figure 1.**
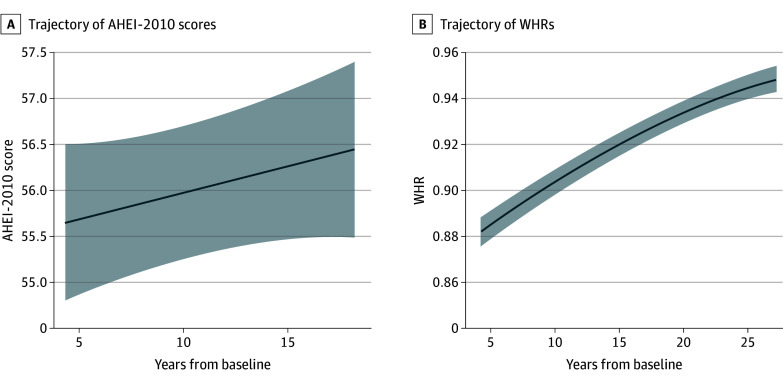
Estimated Longitudinal Trajectories of the Alternative Healthy Eating Index–2010 (AHEI-2010) Score and Waist to Hip Ratio (WHR) A, No significant linear increase in the AHEI-2010 score was found across 11 years (waves 3, 5, and 7) (N = 512 participants). B, There was an increase in WHR (ie, slope) across 21 years (waves 3, 5, 7, 9, and 11) (N = 664 participants), modeled using a cubic spline with *df* = 2. The spline terms ns(t,2)1 and ns(t,2)2 represent the nonlinear relationship over time.

AHEI-2010 scores did not change significantly from wave 3 (β_0_ = 55.41; 95% CI, 54.35-56.47) over the following 11 years (β_1_ = 0.06; 95% CI, –0.02 to 0.13; *P* = .15), indicating consistent diet quality from middle to older age on a group level (Figure 1A). By contrast, WHR increased nonlinearly from wave 3 (β_0_ = 0.88; 95% CI, 0.88-0.89) over the following 21 years (β_1_ = 0.09 [95% CI, 0.08-0.10; *P* < .001]; β_2_ = –0.0001 [95% CI, 0.04-0.05; *P* < .001]) (Figure 1B). The mean outcome measures (white matter metrics, cognitive performance, and hippocampus connectivity maps) are shown in eTable 6 and eFigure 5 in Supplement 1.

### Associations of Diet With Neuroimaging and Cognitive Outcomes

#### AHEI-2010 Intercept

**Figure 2.**
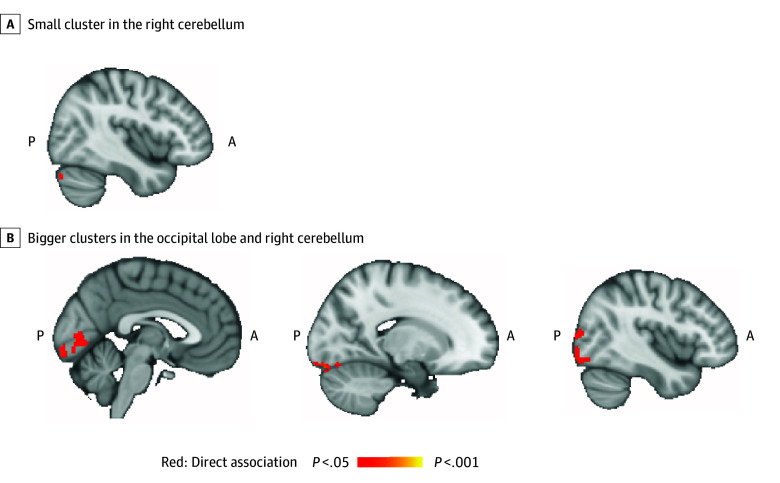
Association Between the Intercept of the Alternative Healthy Eating Index–2010 (AHEI-2010) Score and Hippocampal Functional Connectivity A, Higher functional connectivity of the right hippocampus to a small cluster in the right cerebellum was associated with higher AHEI-2010 scores in midlife (ie, the intercept measured 21 years before the magnetic resonance imaging scan). B, Higher functional connectivity of the left hippocampus to bigger clusters in the occipital lobe and the right cerebellum was associated with higher AHEI-2010 scores in midlife. Images show familywise error–corrected, threshold-free cluster enhancement statistical maps overlaid on a template. A indicates anterior; P, posterior.

There were no associations between the AHEI-2010 intercept (ie, projected midlife diet quality) and white matter microstructure or cognitive performance. Better midlife diet was, however, associated with higher functional connectivity between the left hippocampus and occipital lobe and cerebellum (clusters 1-7; 8-2648 mm^3^; total: 9176 mm^3^; *P* < .05) and 1 small cluster between the right hippocampus and cerebellum (cluster 1; 136 mm^3^; *P* = .04) (Figure 2; eTable 9 and eFigure 7 in Supplement 1).

#### AHEI-2010 Slope

**Figure 3.**
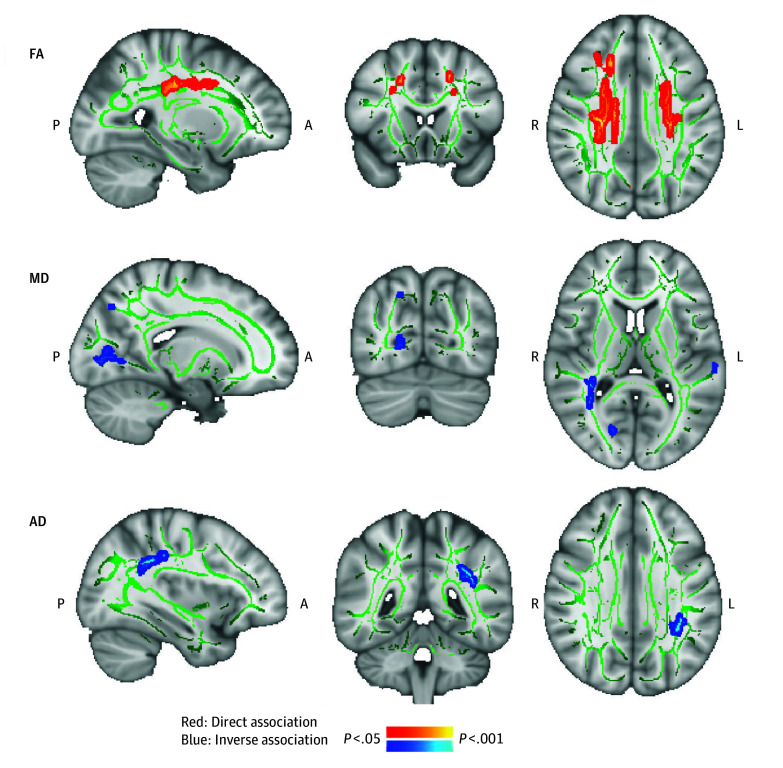
Longitudinal Change in Alternative Healthy Eating Index–2010 (AHEI-2010) Score Associated With White Matter (WM) Connectivity of Fractional Anisotropy (FA), Mean Diffusivity (MD), and Axial Diffusivity (AD) The panels show familywise error–corrected, threshold-free cluster enhancement statistical maps overlaid on a standard image. Green tracts represent the mean FA skeleton. A indicates anterior; L, left; P, posterior; and R, right.

**Figure 4.**
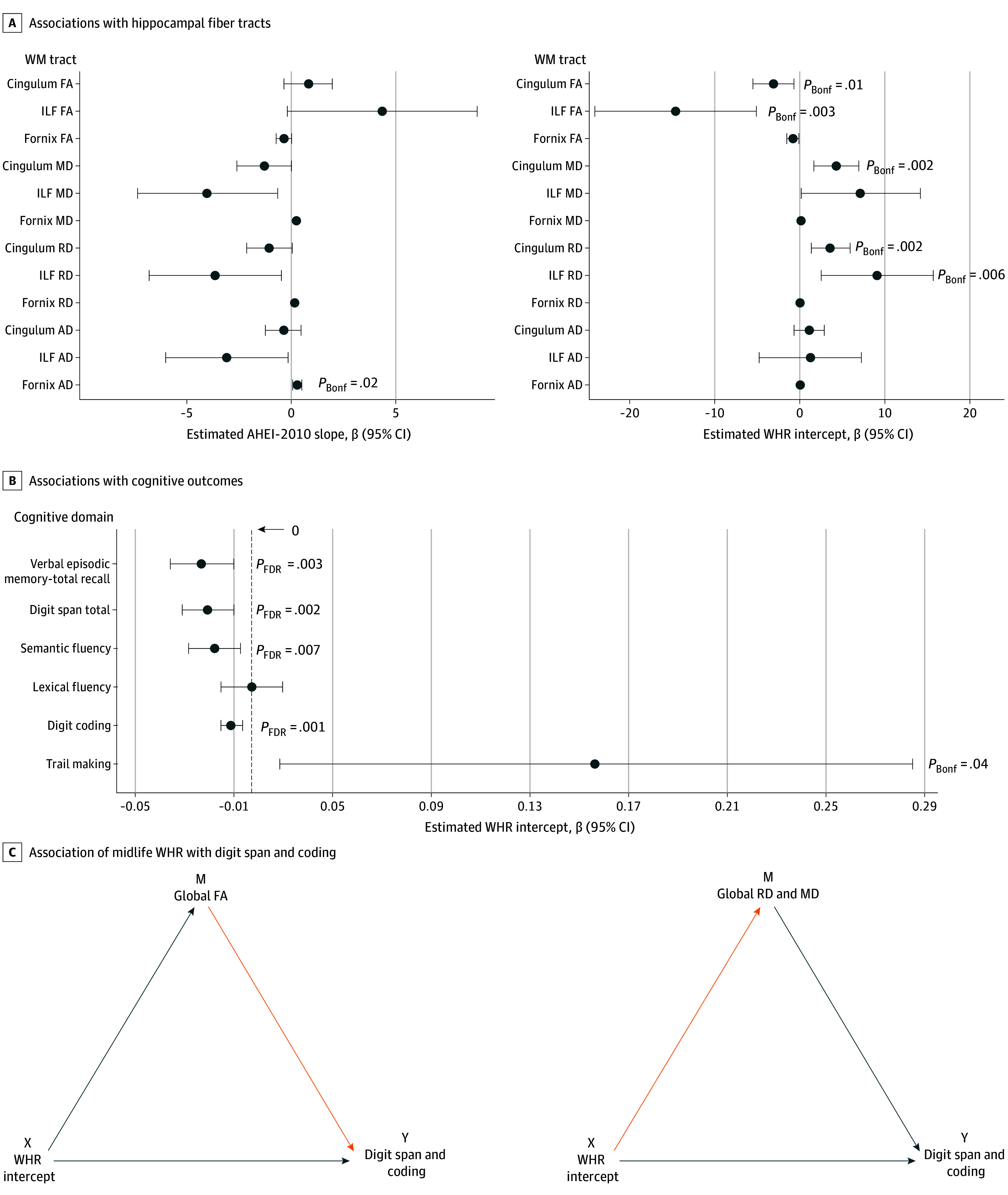
Association Between Alternative Healthy Eating Index–2010 (AHEI-2010) Slope, Waist to Hip Ratio (WHR) Intercept, Hippocampal Fiber Tracts, and Cognitive Performance A, AHEI-2010 slope (n = 506) showed associations with fornix connectivity. WHR intercept (n = 657) was associated with 3 predefined hippocampal white matter (WM) tracts and showed associations between the intercept of WHR with inferior longitudinal fasciculus (ILF) and cingulum connectivity. *P* values are significant Bonferroni-corrected values (*P*_Bonf_). B, Higher WHR intercept was associated with lower cognitive performance on verbal episodic memory, digit span, semantic fluency, digit coding, and trail making. *P* values are significant false discovery rate–adjusted *P* values (*P*_FDR_). C, Causal mediation analysis showed that the WHR in midlife has a direct and indirect association with digit span and digit coding, which was partially mediated by global WM fractional anisotropy (FA), mean diffusivity (MD; for digit coding only), and radial diffusivity (RD). Regression lines are color coded to indicate direct (orange) and inverse (blue) associations. AD indicates axial diffusivity; M, mediator.

Higher AHEI-2010 slopes (ie, individual improvements in diet quality across 11 years) were associated with higher FA (clusters 1-6; 32-8760 mm^3^; total: 19 432 mm^3^; *P* < .05), lower MD (clusters 1-10; 48-2456 mm^3^; total: 5560 mm^3^; *P* < .05), and lower AD (clusters 1-2; 32-2568 mm^3^; total: 2600 mm^3^; *P* < .045) in several white matter tracts (Figure 3; eFigure 6 and eTable 7 in Supplement 1). Higher FA was observed in the corticospinal tract, superior thalamic radiation, frontal aslant tract, and frontal regions. Associations with MD were observed in the optic radiation and the superior parietal lobe (eTable 7 in Supplement 1). A localized association with AD was found in the superior longitudinal fasciculus (SLF). ROI analyses of the 3 predefined hippocampal tracts showed that AD in fornix was associated with AHEI-2010 slopes (β [SE] = 0.26 [0.11]; FDR-corrected *P* = .02 (eTable 8 in Supplement 1; Figure 4A). There were no associations between AHEI-2010 slope and hippocampal functional connectivity. Improvement in diet (higher slope) was associated with better lexical fluency before FDR adjustment (β [SE] = 0.01 [0.00]; FDR-corrected *P* = .21) (eTable 10 in Supplement 1).

As AHEI-2010 scores did not change significantly with age on a group level (ie, slope), we repeated white matter and functional analyses using the mean AHEI-2010 scores across 3 waves for each participant. We found no association between the mean AHEI-2010 score and white matter outcomes. However, higher mean AHEI-2010 scores were associated with higher left hippocampal functional connectivity to clusters in the occipital lobe and right cerebellum (clusters 1-10; 8-1104 mm^3^; total: 2472 mm^3^; *P* < .05) (eFigures 7 and 8 and eTable 9 in Supplement 1).

### Associations of WHR With Neuroimaging and Cognitive Outcomes

#### WHR Intercepts

**Figure 5.**
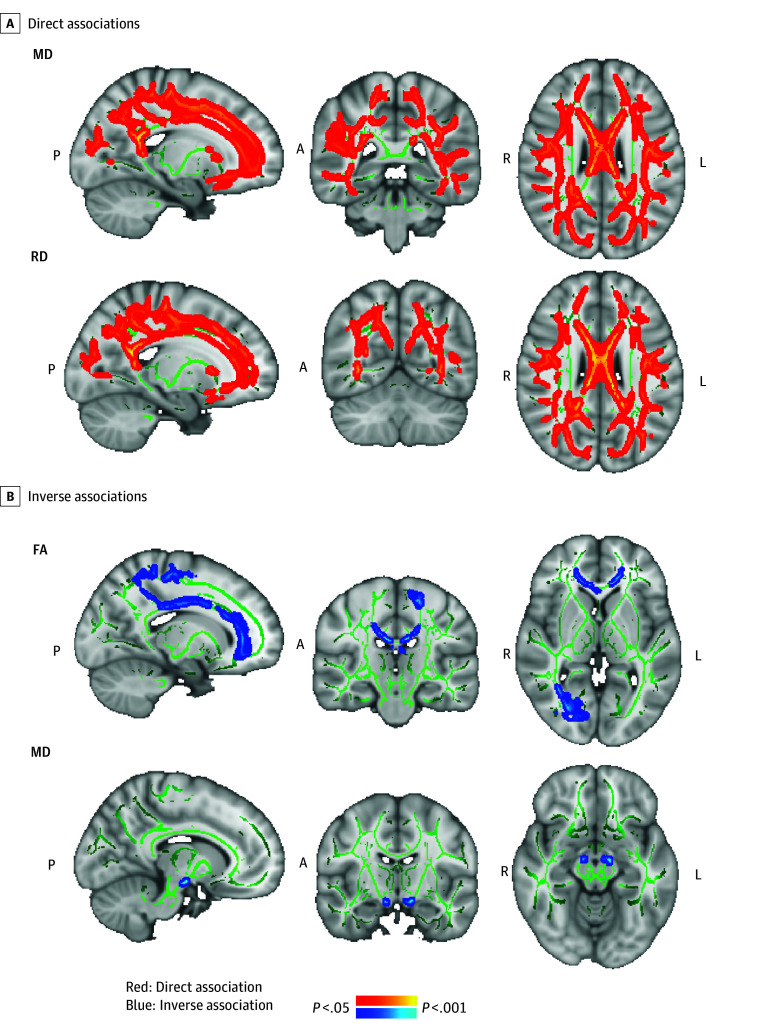
Voxelwise Association Between the Intercept of Waist to Hip Ratio (WHR) and White Matter Connectivity of Mean Diffusivity (MD), Radial Diffusivity (RD), and Fractional Anisotropy (FA) The panels show familywise error–corrected, threshold-free cluster enhancement statistical maps overlaid on a standard image. Green tracts represent the standardized mean FA skeleton. Associations of WHR in midlife (ie, intercept) with MD and RD were direct (A, in red) and with FA and a smaller cluster with MD were inverse (B, in blue). A indicates anterior; L, left; P, posterior; and R, right.

Higher WHR in midlife (ie, higher intercept) was associated with higher MD and RD covering 26.4% (333 088 mm^3^, *P* < .001) and 23.1% (291 888 mm^3^, *P* < .05; clusters 1-15, 48-278 688 mm^3^, total: 291 888 mm^3^, *P* < .05) of the total white matter tracts, respectively, and with lower FA covering 4.9% (clusters 1-23; 8-25 544 mm^3^; total: 61 272 mm^3^; *P* < .05) of the white matter skeleton (Figure 5; eFigure 6 in Supplement 1). The maxima for the direct associations with MD and RD were identified in the cingulum and SLF and ILF (eTable 7 in Supplement 1), whereas the maxima for inverse associations with FA were in the corticospinal tract. We also noted 1 small cluster (0.03% of white matter tract, <50 voxels) showing an inverse association with MD in the corticospinal tract (eFigure 6 in Supplement 1). ROI analyses revealed that higher WHR intercepts were also associated with lower FA and higher RD in the ILF (FA: β [SE] = –14.52 [4.82]; 95% CI, –23.98 to –5.06; FDR-corrected *P* = .003]; RD: β [SE] = 9.11 [3.33]; 95% CI, 2.57-15.65; FDR-corrected *P* = .006]) and with lower FA and higher MD and RD in the cingulum (FA: β [SE] = –3.08 [1.21]; 95% CI, –5.44 to –0.71; FDR-corrected *P* = .011]; MD: β [SE] = 4.28 [1.34]; 95% CI, 1.64-6.92; FDR-corrected *P* = .002]; RD: β [SE] = 3.66 [1.15]; 95% CI, 1.40-5.91; FDR-corrected *P* = .002]) (eTable 8 in Supplement 1; Figure 4A). WHR intercepts were not associated with hippocampal functional connectivity. However, higher intercepts (higher midlife WHR) were associated with lower cognitive performance on the verbal episodic memory (β [SE] = –0.02 [0.01]; 95% CI, –0.03 to –0.01; FDR-corrected *P* = .003), digit span (β [SE] = –0.02 [0.01]; 95% CI, –0.03 to –0.01; FDR-corrected *P* = .002), semantic fluency (β [SE] = –0.02 [0.01]; 95% CI, –0.03 to –0.01; FDR-corrected *P* = .007), digit coding (β [SE] = –0.01 [0.00]; 95% CI, –0.01 to 0.00; FDR-corrected *P* = .001), and trail making (β [SE] = 0.15 [0.07]; 95% CI, 0.01-0.29; FDR-corrected *P* = .04) tests (Figure 4B; eTable 10 in Supplement 1).

#### WHR Slopes

Voxelwise and ROI analyses showed no association between WHR slopes and white matter microstructure or hippocampal functional connectivity. WHR slope was associated with digit span (β [SE] = 0.02 [0.01]; 95% CI, 0.00-0.03; *P* = .03), but this association did not survive FDR adjustment (FDR-corrected *P* = .16) (eTable 10 in Supplement 1).

### Mediation

We examined whether the observed FDR-corrected associations between WHR intercept and cognition (eTable 10 in Supplement 1) were mediated by the MRI variables associated with WHR intercepts (eTables 7 and 8 in Supplement 1). The mediators tested were white matter connectivity measures: global white matter (FA, MD, and RD), ILF (FA and RD), and cingulum (FA, MD, and RD). We found that the association between WHR intercepts and digit span was mediated by global FA (β = –2.96^−03^; 95% CI, –5.56^−03^ to −1.01^−03^; *P* < .001), and global RD (β = –2.69^−03^; 95% CI, –5.03^−03^ to −9.25^−04^; *P* < .001). The association of WHR intercept with digit coding was mediated by global FA (β = –1.33^−03^; 95% CI, –2.38^−03^ to −5.18^−04^; *P* = .002), global RD (β = –1.16^−03^; 95% CI, –2.21^−03^ to −3.26^−04^; *P* = .002), and global MD (β = –8.88^−04^; 95% CI, –1.73^−03^ to −2.40^−04^; *P* = .004) (eFigure 9 and eTable 11 in Supplement 1). Specifically, 0.15 (95% CI, 0.05-0.43; *P* < .001) and 0.14 (95% CI, 0.05-0.35; *P* < .001) of the association of midlife WHR with performance in digit span was mediated by higher global FA and RD, respectively. However, 0.16 (95% CI, 0.06-0.35; *P* = .002), 0.14 (95% CI, 0.03-0.31; *P* = .004), and 0.10 (95% CI, 0.03-0.24; *P* = .004) of the association of midlife WHR with the digit coding performance was mediated by global FA, RD, and MD, respectively. Other significant mediations did not survive the correction for multiple comparisons across the 8 white matter mediators (Bonferroni-corrected *P* < .006) (eTable 11 in Supplement 1).

## Discussion

Findings of this study showed that higher diet quality and lower WHR in midlife (ie, lower intercepts) along with their improved trajectories from middle to older age were associated with structural and functional connectivity of the hippocampus at older ages. Furthermore, lower WHR in midlife (intercept) was associated with better working memory and executive function later in life, and this pathway was mediated by white matter diffusivity. These findings may have implications for optimizing the timing of dietary and metabolic interventions aimed at maintaining brain and cognitive health during the lifespan.

Diet quality remained consistent during the 11-year follow-up, and the mean AHEI-2010 score did not meet the cutoff of a healthy diet at any wave. The mean AHEI-2010 score at baseline (ie, intercept) was 55.6 (95% CI, 54.4-56.5) out of the optimal dietary score of 110 (ie, <80% of the optimal score). The guidelines for similar dietary scores, such as the Healthy Eating Index 2010,^34^ have shown that on a scale of 0 to 100, a score below 50% of the optimal dietary score indicates an overall poor diet quality, whereas scores above 80% indicate a good, healthy diet. Thus, the AHEI-2010 score at baseline indicated generally unhealthy diets among the participants. Previous studies in the Whitehall II Study cohort^16^ have suggested that the lower AHEI-2010 scores may be altered by higher alcohol consumption. However, none of the participants met the criteria for alcohol dependence; thus, it is unlikely that alcohol was a major factor in the results.

Although the group-level dietary quality remained stable during the 11-year study period, we observed that individual-level improvements in diet (higher AHEI-2010 slopes) from middle to older age were directly associated with white matter integrity (higher FA and lower diffusivity). We identified higher FA in widespread tracts (corticospinal tract and superior thalamic radiation), lower MD in the optic radiation and the superior parietal lobe, and lower AD in the SLF. These regions have been implicated as markers for white matter microstructural damage in aging and dementia.^35^ Additionally, the findings are in line with results of studies that higher omega-3 fatty acid levels^6^ and a general healthier diet in older adults are associated with higher FA and lower MD in the SLF and with corpus callosum and higher global FA.^5^ Taken together, we suggest that strategies to improve diet quality and adherence to current dietary guidelines in dementia may benefit white matter microstructure.

Previous studies have found direct associations between better diet quality and larger hippocampal volume,^16,36^ but, to our knowledge, the present longitudinal study is the first to show that better diet quality in midlife (ie, higher AHEI-2010 intercept) is associated with higher hippocampal functional connectivity in older age. We observed higher hippocampal connectivity to the occipital lobe and cerebellum. While these are distant connections of the hippocampus, their volumes have previously been associated with diet.^37,38,39,40^ However, these associations were small, localized, and hence warrant replication.

There are largely consistent reports of associations between high BMI and low white matter integrity from midlife to older age,^5,24,25^ but the associations with more precise measures of abdominal fat, such as WHR, have been less studied. Here, we reported that higher WHR in midlife (intercept) was associated with widespread higher diffusivity of the white matter, affecting up to 23.1% to 26.4% of all white matter tracts. Analyses of hippocampus-associated white matter tracts revealed lower FA and higher diffusivity in the ILF and cingulum. These findings are in line with those of cross-sectional studies showing associations between higher WHR and lower FA in several white matter tracts, including the corpus callosum and ILF in older adults^24^ and cingulum in middle-aged adults.^25^ The ILF and cingulum are known to be implicated in Alzheimer disease,^35^ and our results suggest that these tracts may be especially relevant for WHR-related alterations in axonal and myelin integrity.

Higher abdominal fat in midlife (higher WHR intercept) was also associated with worse cognitive performance in older age across several cognitive domains, including fluency, episodic memory, working memory, and executive function. Previous studies have shown that obesity affects white matter integrity and cognitive performance, such as executive function, in line with previous studies.^24,41^ We also observed that the association of midlife WHR with cognitive performance on the digit span and digit coding tests was mediated by white matter connectivity (global FA and diffusivity). Given that measures of WHR were collected several years before the brain MRI scan, it is reasonable to assume that prior midlife metabolic health (measured as the intercept of WHR) may affect white matter integrity in older age, which, in turn, may have knock-on effects or indirect implications for cognitive performance. This pathway is also likely to be altered by other factors in metabolic health, such as diet, cardiovascular history, blood pressure, cholesterol, and medications, although this study lacked the statistical power to test this using more complex multivariate models. Findings of this study support the prevailing theories of a pathway from lifestyle risk factors to cognitive health via cerebral microstructure.^39^

### Limitations

This study had several limitations. First, dietary data were collected using a semiquantitative FFQ, which can be open to self-report errors. Nevertheless, the FFQ covers a wide range of foods and has been validated by showing the association of nutrients and food consumption with outcomes.^15,42^ Second, diet quality was assessed with the AHEI-2010, which may not be adapted to the dietary habits of all populations; however, the AHEI-2010 score has already been established and used in previous studies.^16^ Third, we adjusted for scanner site using binary measures, as has been standard for the Whitehall II Study cohort^43,44^; however, there are more robust MRI harmonization procedures that could be an alternative approach for future studies in this cohort.^45^ Fourth, statistical analyses were adjusted for sex, but our study was limited by having a predominantly male cohort and lacking the statistical power to examine women and men separately (<20% women). The literature shows sex differences in WHR,^46^ with reported associations of body fat and abdominal fat with cortical thickness in older men but not women.^47^ These sex-specific differences in risk highlight the importance of sex-stratified and balanced studies. Fifth, the cohort was predominantly White British, had high educational levels, was susceptible to survival bias, and therefore was generally healthier than the UK population.^48^ These participant characteristics may limit the generalizability of the study findings.

## Conclusions

In this cohort study of middle-aged to older adults, we found that measures of diet and abdominal fat in midlife (WHR intercepts) were associated with hippocampal functional and white matter connectivity later in life. Higher WHR in midlife was associated with poorer working memory and executive function in older age, and this association was partially mediated by white matter diffusivity. These findings suggest that interventions to improve diet and manage central obesity might be best targeted in midlife (ages 48-70 years) to obtain beneficial outcomes for brain and cognitive health in older age.

## Notes

Supplement 1. **eMethods 1.** MRI Image Acquisition and Pre-processing Details **eMethods 2.** Flowchart of Sample Selection **eTable 1.** Whitehall II Cohort Details and MRI Acquisition Parameters **eFigure 1.** Flowchart of Sample Selection **eMethods 3.** Details of the AHEI-2010 Score **eFigure 2.** Individual Variation in AHEI-2010 Score and WHR Level Trajectories **eMethods 4.** Linear Mixed Effect Model **eTable 2.** Comparison of Likelihoods of Fitted Cubic Spline Linear Mixed Effect With Two or Three Degrees of Freedom (df) for AHEI-2010 Score and the WHR Levels **eTable 3.** Comparison of Likelihoods of Fitted Models (L - Linear and C - Natural Cubic Spine) Linear Mixed Effect Models of AHEI-2010 Score and the WHR Levels **eTable 4.** Summary of Linear Mixed Effect Model Output for Best Fitting Model for AHEI-2010 Score (AHEI - left) and the WHR Levels (Value - right) **eFigure 3.** Linear Mixed Effect Model Diagnostic Plots for AHEI-2010 (A) and WHR (B) **eMethods 5.** Further Details on the Analysis of Structural Connectivity Using DTI and Hippocampal Functional Connectivity Using rsfMRI **eFigure 4.** Hippocampal Seed Mask Used for the Seed-Based Correlation Analysis in Example Subject **eMethods 6.** Mediation Analysis **eMethods 7.** Confounders **eTable 5.** Characteristics of the Participants **eTable 6.** Mean Outcome Measures of Extracted WM Metrics and Cognitive Performance Tests at MRI Scan **eFigure 5.** Mean Functional Connectivity From the Right (A) and Left (B) Hippocampus Mask to the Rest of the Brain **eFigure 6.** AHEI-2010 Slopes and WHR Intercept Associated With WM Connectivity Markers **eTable 7.** Results of the Voxel-Wise Association of AHEI-2010 (n=506) and WHR (n=657) With FA, MD, RD, and AD **eTable 8.** Results of the Region of Interest Analysis Showing Associations of the Fornix, Inferior Longitudinal Fasciculus, and Cingulum With AHEI-2010 Slope (n=506) and WHR Intercept (n=657) **eTable 9.** Cluster Report for Seed-Based Correlation Analysis **eFigure 7.** AHEI-2010 in Midlife (i.e., Intercept) Associated With Functional Hippocampal Connectivity **eFigure 8.** The Association Between the Average AHEI-2010 Score Across Three Waves With Left Hippocampal Functional Connectivity in N=512 Participants **eTable 10.** Associations Between the AHEI-2010 Score (N=506) and WHR (N=657) and Cognitive Outcomes **eTable 11.** Results of 95 % Confidence Intervals of Mediation (1000x Bootstrapping) **eFigure 9.** White Matter Integrity Partially Mediates the Association Between WHR in Midlife and Digit Span and Digit Coding
eReferences

Supplement 2.
Data Sharing Statement
