# Supplementary material for: Association of Diet and Waist-to-Hip Ratio With Brain Connectivity and Memory in Aging
Source: JAMA Netw Open. 2025 Mar 12;8(3):e250171. doi: 10.1001/jamanetworkopen.2025.0171 (PMC11904738; doi:10.1001/jamanetworkopen.2025.0171)
Supplement: Supplement 1. — eMethods 1. MRI Image Acquisition and Pre-processing Details eMethods 2. Flowchart of Sample Selection eTable 1. Whitehall II Cohort Details and MRI Acquisition Parameters eFigure 1. Flowchart of Sample Selection eMethods 3. Details of the AHEI-2010 Score eFigure 2. Individual Variation in AHEI-2010 Score and WHR Level Trajectories eMethods 4. Linear Mixed Effect Model eTable 2. Comparison of Likelihoods of Fitted Cubic Spline Linear Mixed Effect With Two or Three Degrees of Freedom (df) for AHEI-2010 Score and the WHR Levels eTable 3. Comparison of Likelihoods of Fitted Models (L - Linear and C - Natural Cubic Spine) Linear Mixed Effect Models of AHEI-2010 Score and the WHR Levels eTable 4. Summary of Linear Mixed Effect Model Output for Best Fitting Model for AHEI-2010 Score (AHEI - left) and the WHR Levels (Value - right) eFigure 3. Linear Mixed Effect Model Diagnostic Plots for AHEI-2010 (A) and WHR (B) eMethods 5. Further Details on the Analysis of Structural Connectivity Using DTI and Hippocampal Functional Connectivity Using rsfMRI eFigure 4. Hippocampal Seed Mask Used for the Seed-Based Correlation Analysis in Example Subject eMethods 6. Mediation Analysis eMethods 7. Confounders eTable 5. Characteristics of the Participants eTable 6. Mean Outcome Measures of Extracted WM Metrics and Cognitive Performance Tests at MRI Scan eFigure 5. Mean Functional Connectivity From the Right (A) and Left (B) Hippocampus Mask to the Rest of the Brain eFigure 6. AHEI-2010 Slopes and WHR Intercept Associated With WM Connectivity Markers eTable 7. Results of the Voxel-Wise Association of AHEI-2010 (n=506) and WHR (n=657) With FA, MD, RD, and AD eTable 8. Results of the Region of Interest Analysis Showing Associations of the Fornix, Inferior Longitudinal Fasciculus, and Cingulum With AHEI-2010 Slope (n=506) and WHR Intercept (n=657) eTable 9. Cluster Report for Seed-Based Correlation Analysis eFigure 7. AHEI-2010 in Midlife (i.e., Intercept) Associated With Functional Hippocamp [file jamanetwopen-e250171-s001.pdf]

Supplemental Online Content

Jensen DEA, Ebmeier KP, Akbaraly T, et al. Association of Longitudinal Diet and Waist to Hip Ratio With Brain Connectivity and Memory in Aging. *JAMA Netw Open*. Published online March 12, 2025. doi:10.1001/jamanetworkopen.2025.0171

eMethods 1. MRI Image Acquisition and Pre-processing Details
eMethods 2. Flowchart of Sample Selection
eTable 1. Whitehall II Cohort Details and MRI Acquisition Parameters
eFigure 1. Flowchart of Sample Selection
eMethods 3. Details of the AHEI-2010 Score
eFigure 2. Individual Variation in AHEI-2010 Score and WHR Level Trajectories
eMethods 4. Linear Mixed Effect Model
eTable 2. Comparison of Likelihoods of Fitted Cubic Spline Linear Mixed Effect With Two or Three Degrees of Freedom (df) for AHEI-2010 Score and the WHR Levels
eTable 3. Comparison of Likelihoods of Fitted Models (L - Linear and C - Natural Cubic Spine) Linear Mixed Effect Models of AHEI-2010 Score and the WHR Levels
eTable 4. Summary of Linear Mixed Effect Model Output for Best Fitting Model for AHEI-2010 Score (AHEI - left) and the WHR Levels (Value - right)
eFigure 3. Linear Mixed Effect Model Diagnostic Plots for AHEI-2010 (A) and WHR (B)
eMethods 5. Further Details on the Analysis of Structural Connectivity Using DTI and Hippocampal Functional Connectivity Using rsfMRI
eFigure 4. Hippocampal Seed Mask Used for the Seed-Based Correlation Analysis in Example Subject
eMethods 6. Mediation Analysis
eMethods 7. Confounders
eTable 5. Characteristics of the Participants
eTable 6. Mean Outcome Measures of Extracted WM Metrics and Cognitive Performance Tests at MRI Scan
eFigure 5. Mean Functional Connectivity From the Right (A) and Left (B) Hippocampus Mask to the Rest of the Brain
eFigure 6. AHEI-2010 Slopes and WHR Intercept Associated With WM Connectivity Markers
eTable 7. Results of the Voxel-Wise Association of AHEI-2010 (n=506) and WHR (n=657) With FA, MD, RD, and AD
eTable 8. Results of the Region of Interest Analysis Showing Associations of the Fornix, Inferior Longitudinal Fasciculus, and Cingulum With AHEI-2010 Slope (n=506) and WHR Intercept (n=657)
eTable 9. Cluster Report for Seed-Based Correlation Analysis
eFigure 7. AHEI-2010 in Midlife (i.e. Intercept) Associated With Functional Hippocampal Connectivity
eFigure 8. The Association Between the Average AHEI-2010 Score Across Three Waves With Left Hippocampal Functional Connectivity in N=512 Participants

© 2025 Jensen DEA et al. *JAMA Network Open*

eTable 10. Associations Between the AHEI-2010 Score (N=506) and WHR (N=657) and Cognitive Outcomes
eTable 11. Results of 95 % Confidence Intervals of Mediation (1000x Bootstrapping)
eFigure 9. White Matter Integrity Partially Mediates the Association Between WHR in Midlife and Digit Span and Digit Coding
eReferences

This supplemental material has been provided by the authors to give readers additional information about their work.

© 2025 Jensen DEA et al. *JAMA Network Open*

eMethods 1. MRI image acquisition and pre-processing details

The 775 participants of the WHII Imaging Sub-study were scanned on a 3T scanner at the Wellcome Centre for Integrative Neuroimaging (FMRIB Centre, Oxford). Of them, 552 participants were scanned on a 3T Siemens Magnetom Verio scanner (Erlangen, Germany) with a 32-channel head coil (April 2012 - December 2014). After a scanner upgrade, the remaining 223 were acquired using a 3T Siemens Prisma Scanner (Erlangen, Germany) with a 64-channel head–neck coil in the same centre (July 2015-December 2016; detailed protocol in [1]). The scan parameters were identical or closely matched between scanners, and a scanner model covariate was used in all analyses (eTable 1).

eTable 1: Whitehall II cohort details and MRI acquisition parameters.

**Cohort**	**Whitehall II cohort (WHII)**	
**Study design**	Cross-sectional and longitudinal diet/WHR data	
**Healthy Participants**	N=775	
**Age range**	60-85 years (Wave 11)	
**Scanner**	3T - Verio	3T - Prisma
**Structural MRI**		
**T1-sequence**	ME-MPRAGE	MPRAGE
**TR (ms)**	2,530	1,900
**TE (ms)**	1.79/3.65/5.51/7.37	3.97
**TI (ms)**	1,380	904
**Flip angle**	7°	8°
**Voxel dimension (mm^3)**	1x1x1	1x1x1
**Field of view (mm)**	256	192
**Acquisition time (min:sec)**	6:12	5:31
**Resting-state fMRI**		
**Sequence**	Multiband	
**TR (ms)**	1300	
**TE (ms)**	40	91
**Flip angle**	66°	
**Voxel dimension (mm^3)**	2x2x2	
**Field of view (mm)**	212	
**Number of volumes**	460	
**Acquisition time (min:sec)**	10:10	

Abbreviations: T - Tesla, TR - repetition time, TE - echo time, TI - inversion time, ME-MPRAGE - multi-echo magnetization-prepared rapid acquisition with gradient echo, WHR - wait-to-hip ratio.

T1-weighted structural MRI (multi-echo MPRAGE sequence with motion correction, TR=2530ms, TE=1.79/3.65/5.51/7.37ms, flip angle=7°, FOV=256 mm, voxel dimension=1mm isotropic, acquisition time=6min 12s), multiband echo-planar imaging resting-state fMRI scans (voxel=2mm isotropic, TR=1.3s, acquisition time=10min 10s, multi-slice acceleration factor=6, number of volumes=460) and diffusion-weighted images using an echo planar sequence, with 60 diffusion-weighted directions (*b*-value=1500s/mm^2), 5 non-diffusion weighted images (*b*-value=0s/mm^2) and one B0 volume in the reversed phase-encoded direction (TR=8900ms, TE=91.2ms, FOV=192mm, voxel dimension=2mm isotropic) were analysed for this study.

MRI data was pre-processed using FSL version 6.0.5 [2, 3] as described in Filippini & collegues [1]. Bias correction using FSL-ANAT, brain extraction, and partial-volume tissue segmentation using FMRIB Automated Segmentation Tool (*FAST*, [4]) were performed on T1 scans. Resting-state fMRI data were pre-processed using motion correction, brain extraction, high-pass temporal filtering, and field-map correction tools in FEAT. Non-neuronal fluctuations were regressed out of the 'signal' using single-subject independent component analysis (ICA) and automatic component classification using FMRIB's ICA-based X-noiseifier (*FIX*, [5, 6]). Diffusion-weighted scans were pre-processed using the FMRIB diffusion toolbox (*FDT*), which included

motion correction and correction for eddy currents with *FSL-TOPUP* [7]. Diffusivity and anisotropy maps were extracted using *DTIfit* and aligned into standard space using FMRIB's Nonlinear Registration Tool (*FNIRT*).

eMethods 2. Flowchart of sample selection

We included participants of the Imaging Sub-study who had information on diet from at least one previous wave, WHR from at least two previous waves and good quality structural, rsfMRI and DTI scans (i.e., no excessive head motion artefacts or blurry images, or incidental findings such as large strokes, tumours, or brain cysts). The final sample for the fMRI analysis consisted of n=665 and n=512 participants for analyses of WHR and diet respectively (eFigure 1).

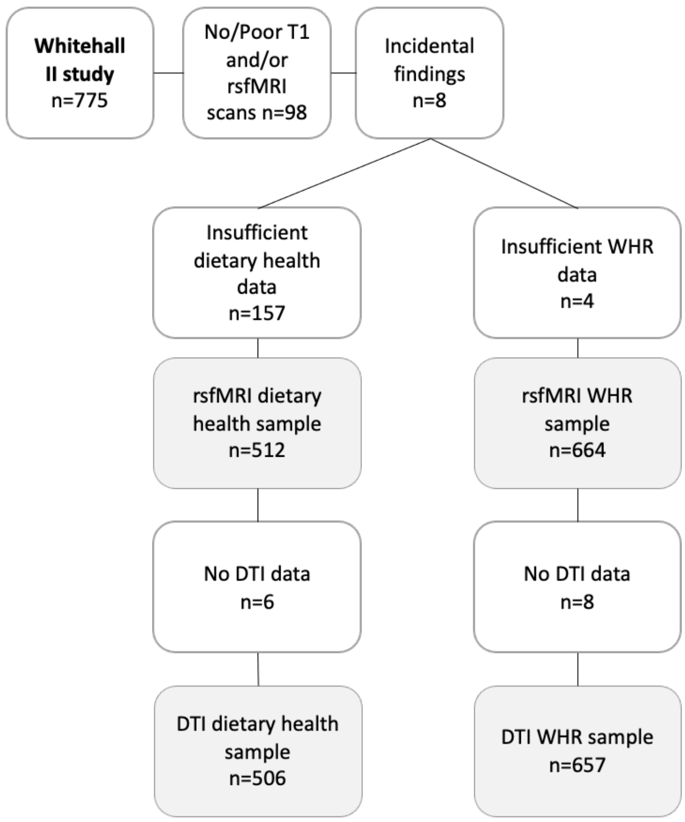

eFigure 1: Flowchart of sample selection.

eMethods 3. Details of the AHEI-2010 score

The AHEI-2010 score [8] is based on eleven components, where a higher score is related to a higher intake of vegetables, fruit, whole grains, nuts, legumes, long chain ω-3 fats, and polyunsaturated fatty acids, avoidance or low intake of sugar-sweetened drinks and fruit juice, red and processed meat, trans-fat, sodium and avoidance or low consumption of alcohol. Each component was scored from 0 to 10 points, where a score of 10 points indicated that the recommendations were fully met and a score of 0 represented the least healthy dietary behaviour. All the component scores were summed to obtain the total AHEI-2010 score [8].

1. Individual trajectories

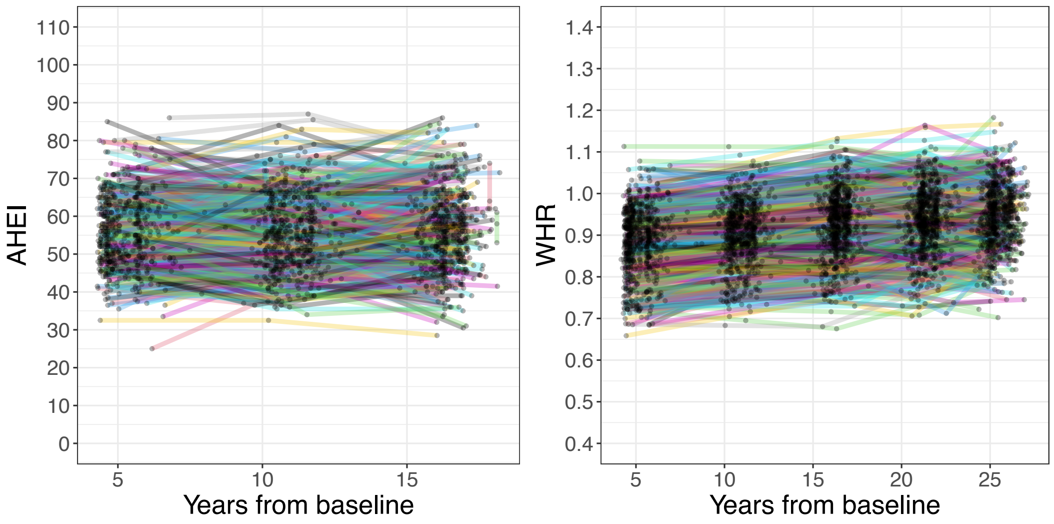

eFigure 2: Individual variation in AHEI-2010 score and WHR level trajectories.

eMethods 4. Linear Mixed Effect Model

As dependent variables in the linear mixed effect model with maximum likelihood estimation (LME function, *NLME* package in R version 4.2.1), we used WHR values from five waves (21 years) and the dietary AHEI-2010 score [8] over three waves (11 years). We allowed for correlations between time points by modelling the errors using a continuous autoregressive process, rather than applying it directly to a continuous time covariate. This approach captures the temporal correlation structure in the residuals.

We tested whether the addition of a natural cubic spline (also known as restricted cubic spline) term for time improved model fit (see further information on cubic spline model in [9]). In short, a natural cubic spline consists of cubic polynomials that ensure continuity and smoothness at each knot, with an additional constraint of linearity at the ends of the curve, typically before the first and after the last knot. A linear mixed-effect model incorporating a natural cubic spline function *b(t)* with *K* knots includes a linearity constraint for values $t < \xi_1$ and $t > \xi_K$. We estimated the degrees of freedom used in the R function *ns() (R package lspline,* [10]) by comparing the model fit (using AIC/BIC and visually i.e. using the QQ and residual plot) between a model with df=2 and the next higher model. If the model fit was significantly better in next higher df model, we chose that model to compare its model fit with the linear model. For the model with df=2, a natural cubic spline for age (measured from Wave 3 to Wave 11) with 1 knot, invokes 2 cubic polynomials. In each linear mixed effect model, we employed the following equations for each dependent variable:

1) Linear Model

$$val_n = \beta_0 + \beta_1 t_n + e_n$$

2) Natural Cubic Spline Model

$$val_n = \beta_0 + \beta_1 t_n + \sum_{k=1}^{K-2} b_{ki} (\beta_2 t_n^2, -, \xi_k)_*^2 + e_n$$

val = WHR/diet value is the dependent variable for each participant (n) at each timepoint
$\beta_0 = random\ intercept$
$\beta_1 = random\ linear\ slope$
$b(t) = cubic\ spline\ function$
$K = knots$
t = time from baseline (Wave 3) for each wave, t^2 *is the squared time variable*
e = is the residual error.

© 2025 Jensen DEA et al. *JAMA Network Open*

In the following we present the comparison of likelihoods of fitted models:
To estimate the degrees of freedom (df) for the cubic spline linear mixed effect model, we compared model with df of 2 with a model with df of 3. If the model fit was significantly improved, we used a cubic spline model with df 3, but otherwise a model with df of 2. For AHEI-2010 and WHR a cubic spline linear mixed effect model with df of 2 has a significantly better model fit compared to the model with df of 3.

eTable 2: Comparison of likelihoods of fitted cubic spline linear mixed effect with two or three degrees of freedom (df) for AHEI-2010 score and the WHR levels.

	Model	df	AIC	BIC	*Log-Likelihood*	Test	Likelihood ratio	p-value
**WHR**	C df=2	8	-11847.35	-11798.49	5931.674			
	C df=2	9	-11845.49	-11790.52	5931.743	1 vs 2	0.1390025	0.7093
**AHEI-2010**	C df=2	8	10842.97	10885.67	-5413.487			
	C df=3	9	10844.76	10892.79	-5413.381	1 vs 2	0.212314	0.645

Abbreviations: df - degree of freedom, AIC - Akaike's Information Criteria, BIC - Bayesian Information Criteria, L - linear mixed effect model, C - cubic spline linear mixed effect model, AHEI-2010 - Alternative Health Eating Index 2010.

eTable 3: **Comparison of likelihoods of fitted models (L-linear and C-natural cubic spine) linear mixed effect models of AHEI-2010 score and the WHR levels.**

In WHR, but not the AHEI-2010 model, the cubic spline significantly improved the model fit.

	Model	df	AIC	BIC	*Log-Likelihood*	Test	Likelihood ratio	p-value
**WHR**	L	7	-11817.70	-11774.95	5915.852			
	C df=2	8	-11847.35	-11798.49	5931.674	1 vs 2	31.64404	<0.0001
**AHEI-2010**	L	7	10841.04	10878.40	-5413.521			
	C df=2	8	10842.97	10885.67	-5413.487	1 vs 2	0.06903869	0.7927

Abbreviations: df - degree of freedom, AIC - Akaike's Information Criteria, BIC - Bayesian Information Criteria, L - linear mixed effect model, C - cubic spline linear mixed effect model, AHEI-2010 - Alternative Health Eating Index 2010.

The comparison between the model fits revealed that a linear mixed effect model for fitting the trajectory of AHEI-2010 score with age, but a linear model with cubic splines and df=2 for fitting the WHR trajectory with age were the best fitting models (see eTable 4 and eFigure 3).

© 2025 Jensen DEA et al. *JAMA Network Open*

eTable 4: Summary of linear mixed effect model output for best fitting model for AHEI-2010 score (ahei - left) and the WHR levels (value - right).

Summary output of linear mixed effect models

	ahei				value			
Predictors	*Estimates*	*CI*	*p-value*	*df*	*Estimates*	*CI*	*p-value*	*df*
Intercept	55.41 (0.54)	54.35 – 56.47	**<0.001**	1023.00	0.88 (0.00)	0.88 – 0.89	**<0.001**	2654.00
t	0.06 (0.04)	-0.02 – 0.13	0.147	1023.00				
ns(t, 2)1					0.09 (0.00)	0.08 – 0.10	**<0.001**	2654.00
ns(t, 2)2					0.05 (0.00)	0.04 – 0.05	**<0.001**	2654.00
**Random Effects**								
σ^2	31.10				0.00			
τ_{00}	77.19 $_{oxf\_id}$				0.01 $_{oxf\_id}$			
τ_{11}	0.28 $_{oxf\_id.t}$				0.00 $_{oxf\_id.t}$			
ρ_{01}	-0.51 $_{oxf\_id}$				-0.53 $_{oxf\_id}$			
ICC	0.67				0.91			
N	512 $_{oxf\_id}$				664 $_{oxf\_id}$			
Observations	1536				3320			
Marginal R^2 / Conditional R^2	0.001 / 0.674				0.061 / 0.912			
AIC	10841.042				-11847.347			

© 2025 Jensen DEA et al. *JAMA Network Open*

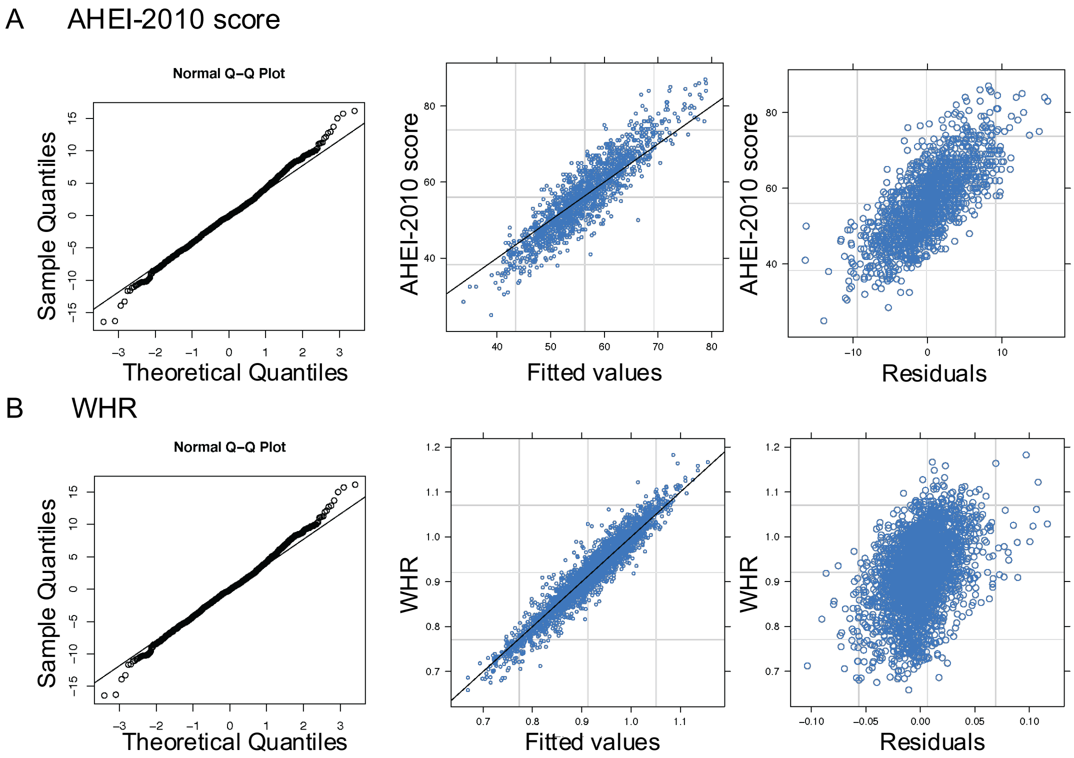

eFigure 3: Linear mixed effect model diagnostic plots for AHEI-2010 (A) and WHR (B).

Each panel includes three plots arranged from left to right: the Q-Q plot, which assesses the normality of residuals; the fitted values plot, illustrating the relationship between observed and predicted values; and the residuals plot, which evaluates the homoscedasticity and independence of residuals. These plots collectively provide insights into the model fit and assumptions for each outcome variable.

eMethods 5. Further details on the analysis of structural connectivity using DTI and hippocampal functional connectivity using rsfMRI

A. Hippocampal structural connectivity using DTI

WM microstructure was assessed using DTI scans analysed with tract-based spatial statistics (*FSL-TBSS)*[11]. DTI detects the directionality of diffusion of water molecules within the axons. This diffusion is unrestricted along the axon, hindered perpendicularly due to the presence of the myelin sheath. The directionality and diffusivity were qualified by DTI parameters such as fractional anisotropy (FA), radial diffusivity (RD), axial diffusivity (AD), and mean diffusivity (MD). If the diffusion in a voxel is anisotropic this means that it follows more easily along the axons as perpendicular to them. These DTI parameters give indirect indicators of fibre tract integrity and have been found to differentiate between mild cognitive impairment, Alzheimer's disease, and other dementias (see review [12]).

Global FA, MD, RD, and AD were extracted from the mean TBSS skeleton, with FA values ranging from 0 (representing isotropic diffusion) to 1 (representing anisotropic diffusion). The mean TBSS skeleton is derived from a mean FA image, which is created by averaging the aligned FA images of all subjects. This mean FA image is then thinned through a process called skeletonization, which involves identifying the local maxima of FA values perpendicular to the tract direction, ensuring that the skeleton accurately represents the centers of the tracts.

We additionally extracted FA, MD, RD, and AD values from three regions of interest in proximity to the hippocampus based on the literature [13]: the fornix, the inferior longitudinal fasciculus (ILF), and the cingulum.

B. Hippocampal functional connectivity using rsfMRI

Hippocampal functional connectivity was analysed using seed-based correlation analyses. For the right and left hippocampus masks obtained from FreeSurfer 6.0 [14] (version 6.0), we created a binarised seed mask and applied smoothing using a kernel sphere with a radius of 4mm, and applied a threshold of 0.75 in structural space (see eFigure 4). Then, each hippocampus mask was registered from structural to resting-state fMRI space using the FSL *applywarp* command.

For each subject, we used *first-level FEAT* analyses to calculate seed-based connectivity maps of both the right and left hippocampus to the rest of the brain. Within each *first-level FEAT*, a General Linear Model (GLM) was fitted to the data. In each GLM, we used two contrasts: one to determine brain regions positively correlated with the seed and one for negative correlations. As we were interested in the connectivity of hippocampus seed with grey matter, we regressed out the signal from white matter and cerebrospinal fluid (CSF) using white matter and the CSF time series as confounders in the *first-level FEAT* for each subject. This produced a hippocampal partial correlation (or functional connectivity) map for each subject, which shows regions that are significantly positively and negatively correlated with the left and right hippocampus.

To identify between-subject differences, individual functional connectivity maps were used for the *higher-level FEAT* analysis. We created GLMs for (1) the intercept, (2) the linear slope (for WHR and AHEI-2010), and (3) the quadratic slope (for WHR only). Between-subject differences were investigated using nonparametric permutation inference (FSL's tool *randomise,* [15]) with 1000 permutations. Analyses were restricted to voxels within the grey matter (using the MNI152 template thresholded to 0.3). A 4D covariate image was generated to obtain grey matter density masks using the command *feat_gm_prepare* in FSL [16]. This grey matter density image was used as a voxel-dependent confounder (in addition to all other confounders listed, see Methods in 2.7).

The group-level voxel-wise analysis included strict threshold-free cluster enhancement (*TFCE*) and a correction for family-wise errors (*FWE*, $p<0.05$) for multiple voxel-wise comparisons. Mean hippocampal connectivity values were extracted from significant clusters and plotted for visualisation.

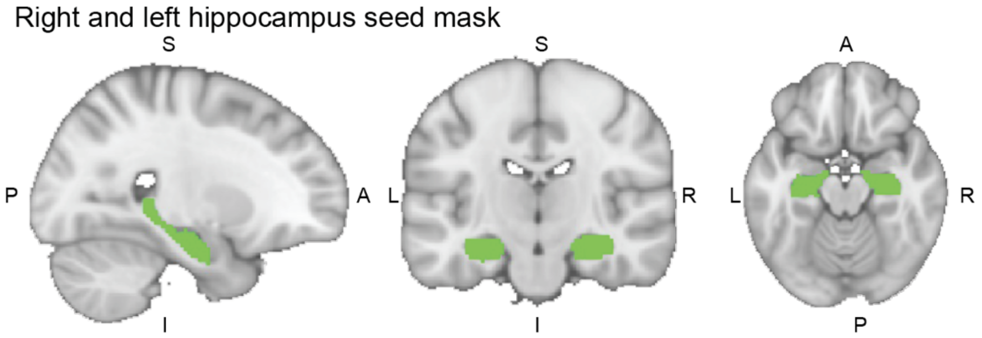

eFigure 4: Hippocampal seed mask used for the seed-based correlation analysis in example subject.

MNI coordinates in this view: x=33, y=54, z=27.

eMethods 6. Mediation Analysis

We performed a causal mediation analysis using the *mediation* package in R to test whether the association of WHR (intercept and slope across 21 years) and dietary quality (i.e., AHEI-2010 intercept and slope across 11 years) on cognitive performance (working memory, fluency, and executive function) at the MRI Phase was mediated by MRI markers (rsfMRI and DTI).

This was used to test the statistical significance of the direct effect from the indirect (i.e., mediated) effect of diet/WHR on cognitive performance. Thus, diet/WHR intercept/slope was set as the X-variable, MRI variables were set as mediator (M) variables, and the cognitive performance measures were set as outcome variables (Y).

The mediation analysis was performed on participants with complete data only when there was a significant association between X and Y. We ran two separate multiple linear models to assess the individual path of the indirect effect. The first model had cognitive performance as the dependent variable (Y), and the brain MRI variable as the mediator variable (M), and other covariates (see below) as independent variables (IVs; Path: M + covariates → Y). In the second model, M was the dependent variable and diet/WHR intercept/slope + the independent variables (Path: X + covariates → M). If both the individual paths were significant, we ran the causal

mediation analysis (Path: X → M → Y) using nonparametric bootstrapping to generate 95% confidence intervals (CIs) with 1,000 simulations. Therefore, we tested mediations only if there was a significant direct effect of the intercept and slopes of AHEI-2010/WHR with cognitive outcomes (X→Y). In that case, we then tested those brain connectivity mediators (M) that were significantly associated with AHEI-2010/WHR (X→M).

eMethods 7. Confounders

All analyses were adjusted for sex, MRI scanner model, age, years of education, , physical activity, and the Montreal Cognitive Assessment (MoCA) score all measured at the MRI timepoint. Physical activity was measured using the Community Healthy Activities Model Program for Seniors (CHAMPS) questionnaire [17]. This self-reported questionnaire assesses the weekly frequency and duration of various activities, and the metabolic equivalent of task (met)/week of all activities was used as a confounder. Additional adjusting for the smoking status ("Yes", "Occasional smoker", "No") at MRI timepoint was not done because of the very low numbers of smokers.

RsfMRI analyses were additionally corrected for head motion and the voxel-wise grey matter density confounder. To quantify the quality instead of the quantity of food intake we included the total energy intake (kcal/day) as a confounder for the AHEI-2010 analyses. Moreover, we included BMI as a confounder for AHEI-2010 analyses to assess diet quality unrelated to body composition.

© 2025 Jensen DEA et al. *JAMA Network Open*

eResults

1. Participants characteristics

*eTable 5: Characteristics of the participants**

	Wave 3 1991-1994	Wave 5 1997-1999	Wave 7 2002-2004	Wave 9 2007-2009	Wave 11 2011-2013	MRI scan 2012-2016
**AHEI-2010 sample included in functional connectivity analyses**						
N (% female, % male)	512 (21.29%, 78.71%)					
Age (years)	47.79 ± 5.19	53.57 ± 5.16	59.03 ± 5.14	..	..	69.81 ± 5.11
AHEI-2010 score. (all)<br>(women)<br>(men)	55.61 ± 9.44<br>56.73 ± 9.43<br>55.24 ± 9.41	56.19 ± 9.66<br>56.75 ± 9.93<br>56.02 ± 9.66	56.25 ± 10.27<br>56.98 ± 10.32<br>56.01 ± 10.20	..	..	..
Education (years)	..	..	..	..	..	14.01 ± 3.08
Physical activity (met/week)	..	..	..	..	..	2748.46 ± 1822.31
Mean energy intake	2202.59 ± 504.43 (Average across waves 3,5, and 7)					..
BMI	..	..	..	..	..	26.24 ± 4.13
MoCA	..	..	..	..	..	27.15 ± 2.40
Smoking status (smoker/ occasional smoker/ no smoker)						11/1/500
Ethnicity (white/ non-white)						481/31
**WHR sample included in functional connectivity analyses**						
N (% female, % male)	664 (19.88%, 80.12%)					
Age (years)	47.71 ± 5.14	53.47 ± 5.12	58.96 ± 5.09	63.88 ± 5.10	67.94 ± 5.10	69.75 ± 5.07
WHR (all)<br>(women)<br>(men)	0.89 ± 0.08<br>0.89 ± 0.08<br>0.89 ± 0.08	0.91 ± 0.07<br>0.91 ± 0.07<br>0.91 ± 0.07	0.93 ± 0.08<br>0.93 ± 0.08<br>0.93 ± 0.08	0.93 ± 0.07<br>0.94 ± 0.07<br>0.93 ± 0.08	0.95 ± 0.07<br>0.95 ± 0.07<br>0.95 ± 0.07	..
Education (years)	..	..	..	..	..	14.11 ± 3.11
Physical activity (met/week)	..	..	..	..	..	2750.70 ± 1810.21
BMI	..	..	..	..	..	26.07 ± 4.14
MoCA	..	..	..	..	..	27.23 ± 2.28
Smoking status (smoker/ occasional smoker/ no smoker)						18/4/642
Ethnicity (white/ non-white)						630/34

© 2025 Jensen DEA et al. *JAMA Network Open*

Abbreviations: AHEI-2010 - Alternative-Health Eating Index 2010, WHR - waist-to-hip ratio, met- Metabolic Equivalent of Task, MoCA - Montreal Cognitive Assessment.
**Measures are specified as their mean ± their standard deviation. Diet was measured using the AHEI-2010 score across three waves (3,5, and 7) and an MRI scan was acquired in N=512 participants 11 years later. Waist-to-hip ratio (WHR) was measured across 21-years in five waves (3, 5, 7, 9, and 11) and the MRI scan was acquired in N=664 participants.*

2. Mean WM metrics and cognitive tests measurements

eTable 6: Mean outcome measures of extracted WM metrics and cognitive performance tests at MRI scan.

Measures are specified as their mean ± their standard deviation. Diffusivity measures (MD, RD, AD) are multiplied with 10^3.

	AHEI-2010 sample included in functional connectivity analyses	AHEI-2010 sample included in WM connectivity analyses	WHR sample included in in functional connectivity analyses	WHR sample included in WM connectivity analyses
N (% female)	512 (21.29%)	506 (21.54%)	664 (19.88%)	657 (20.09%)
Age (years)	69.81 ± 5.11	69.78 ± 5.10	69.75 ± 5.07	69.72 ± 5.06
Working memory (verbal episodic memory - total recall)	27.49 ± 4.68	27.52 ± 4.66	27.54 ± 4.60	27.57 ± 4.58
Working memory (digit span total)	30.63 ± 5.71	30.67 ± 5.71	30.79 ± 5.70	30.82 ± 5.69
Semantic fluency	22.49 ± 5.63	22.49 ± 5.64	22.34 ± 5.47	22.35 ± 5.47
Lexical fluency	15.75 ± 4.54	15.72 ± 4.55	15.77 ± 4.49	15.76 ± 4.49
Executive function (digit coding)	62.65 ± 13.29	62.74 ± 13.23	63.07 ± 13.21	63.14 ± 13.17
Executive function (trail making)*	0.59 ± 0.48	0.58 ± 0.47	0.59 ± 0.45	0.58 ± 0.44
WM global FA		0.48 ± 0.02		0.29 ± 0.01
WM global MD		0.68 ± 0.03		0.36 ± 0.01
WM global RD		0.49 ± 0.03		0.28 ± 0.02
WM global AD		1.08 ± 0.02		0.64 ± 0.01
WM ILF FA		0.14 ± 0.01		0.14 ± 0.01
WM Cingulum FA		0.57 ± 0.03		0.58 ± 0.03
WM Fornix FA		0.37 ± 0.09		0.37 ± 0.09
WM ILF MD		0.19 ± 0.01		0.19 ± 0.01
WM Cingulum MD		0.63 ± 0.03		0.63 ± 0.03
WM Fornix MD		1.38 ± 0.22		1.43 ± 0.26
WM ILF RD		0.13 ± 0.01		0.13 ± 0.01
WM Cingulum RD		0.37 ± 0.03		0.38 ± 0.03
WM Fornix RD		1.09 ± 0.27		1.14 ± 0.31
WM ILF AD		0.31 ± 0.01		0.32 ± 0.01
WM Cingulum AD		1.14 ± 0.04		1.15 ± 0.04
WM Fornix AD		1.96 ± 0.15		2.0 ± 0.16

Abbreviations: AHEI-2010 - Alternative-Health Eating Index 2010, WHR - waist-to-hip ratio, ILF - inferior longitudinal fasciculus, WM - white matter, FA - fractional anisotropy, MD - mean diffusivity, RD - radial diffusivity.
** lower values indicate higher performance*

© 2025 Jensen DEA et al. *JAMA Network Open*

3. Mean hippocampus connectivity maps

Across N=664 participants (i.e. participants of the WHR analyses), the right and left hippocampus showed strong correlations to each other, and to areas of the default mode network (DMN) such as the medial prefrontal cortex, posterior cingulate cortex/precuneus, and angular gyrus (see eFigure 5). We observed high hippocampal functional connectivity with most other cortical areas (r>0.3), whereas the functional connectivity to the brainstem and other subcortical structures was lower (r<0.2).

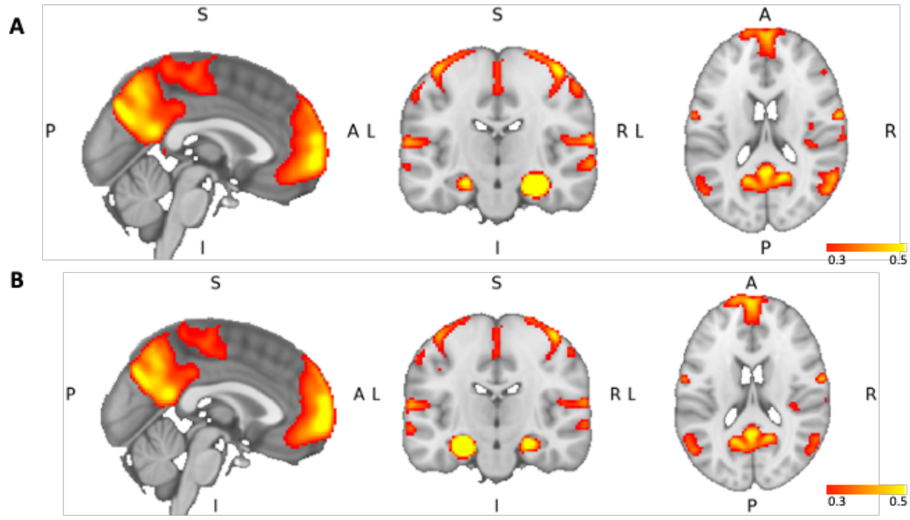

eFigure 5: Mean functional connectivity from the right (A) and left (B) hippocampus mask to the rest of the brain.

The intensity mask is thresholded to 0.3 for the lower limit and 0.5 for the upper limit. Maps are overlaid with the MNI-152 template. MNI coordinates are x=0, y=-18, z=18.

4. Structural connectivity outcomes

4.1 Results of the voxel-wise association of AHEI-2010 and WHR with WM

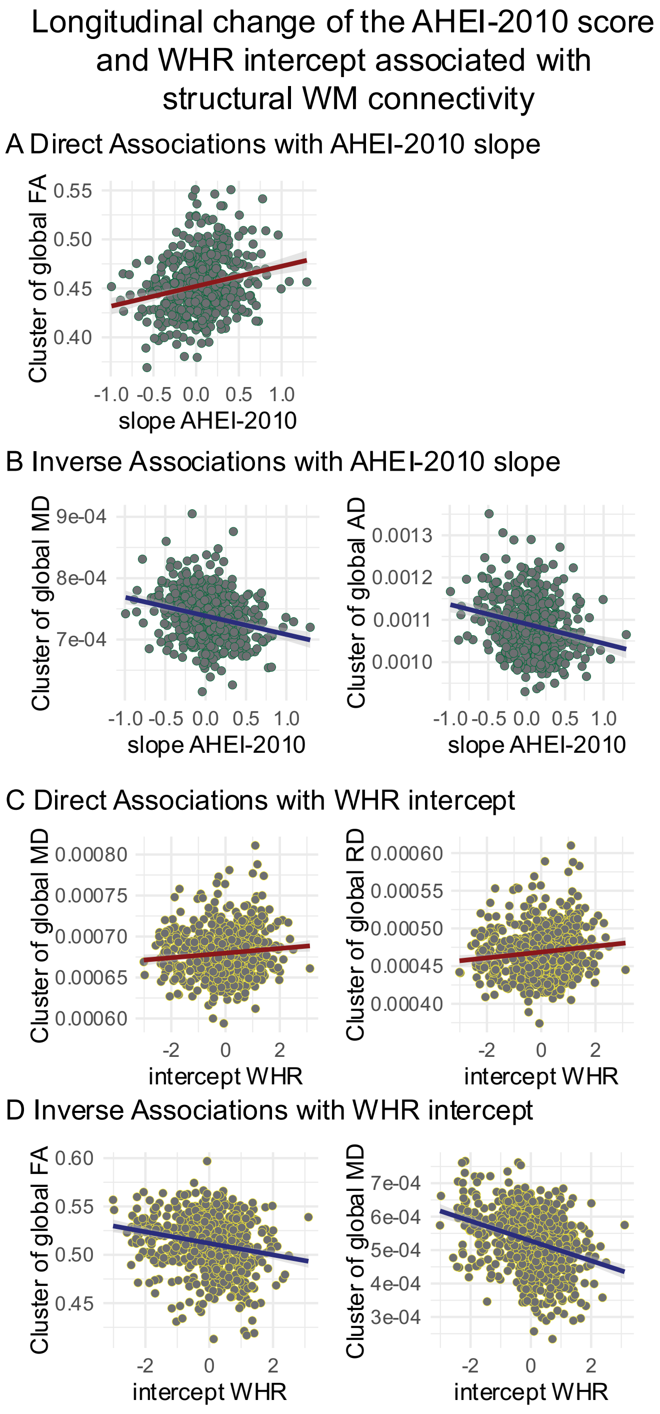

eFigure 6: AHEI-2010 slopes and WHR intercept associated with WM connectivity markers.

Direct (A, C, red linear regression line) and Inverse (B, D, blue linear regression line) associations of the association between WM connectivity parameter estimates and AHEI-2010 slope (A, B, green outline) and WHR intercept (C, D, yellow outline).

© 2025 Jensen DEA et al. *JAMA Network Open*

*eTable 7: Results of the voxel-wise association of AHEI-2010 (n=506) and WHR (n=657) with FA, MD, RD, and AD.**

Clusters larger than 500 voxels are highlighted in bold.

Outcome (association)	Cluster size (mm^3)	Cluster number	Number of voxels	p-value at maxima	Coordinates of maxima (x y z)	Location of maxima
**AHEI-2010 (slope)**						
**FA (direct)**	**19432**	**1**	**1095**	**0.024**	**28 -32 32**	**R Superior Longitudinal Fasciculus**
		**2**	**671**	**0.028**	**-23 -9 34**	**R Superior corona radiata**
		**3**	**500**	**0.039**	**27 24 26**	**R Anterior thalamic radiation**
		4	152	0.047	17 -16 35	Corpus Callosum
		5	7	0.05	-24 19 30	L Superior corona radiata
		6	4	0.048	5 -70 34	Parietal Cortex
**MD (inverse)**	**5560**	1	307	0.046	36 -42 6	R Posterior Thalamic Radiation
		2	151	0.04	18 -77 9	R Optic radiation
		3	90	0.049	35 -5 -12	R Uncinate fasciculus
		4	57	0.049	25 -67 1	R Optic radiation
		5	35	0.05	39 -5 -23	R Inferior Longitudinal Fasciculus
		6	19	0.05	43 -18 -15	R Inferior Longitudinal Fasciculus
		7	15	0.034	-61 -24 11	L Temporal Cortex
		8	8	0.05	43 -16 -19	R Inferior Longitudinal Fasciculus
		9	7	0.043	18 -73 43	R Parietal Cortex
		10	6	0.05	-32 -47 25	L Optic Radiation
**AD (inverse)**	**2600**	1	321	0.033	-34 -44 31	L Superior Longitudinal Fasciculus
		2	4	0.045	18 -73 43	R Superior Parietal Lobe
**WHR (intercept)**						
**FA (inverse)**	**61272**	**1**	**3193**	**0.031**	**23 -88 0**	**R Inferior Fronto-occipital Fasciculus**
		**2**	**2615**	**0.034**	**16 -1 35**	**Corpus Callosum**
		**3**	**1166**	**0.044**	**-17 -37 61**	**L Corticospinal Tract**
		4	144	0.013	28 -65 -35	R Cerebellum
		5	115	0.04	-4 -20 17	Fornix, L Anterior thalamic radiation
		6	92	0.049	35 -66 -4	R Optic Radiation
		7	87	0.048	-13 -46 44	L Cingulum
		8	52	0.048	44 -63 -7	R Superior Longitudinal Fasciculus (temporal part)
		9	46	0.044	-21 -71 -32	L Cerebellum

© 2025 Jensen DEA et al. *JAMA Network Open*

eTable 7 (continued)

Outcome (association)	Cluster size (mm³)	Cluster number	Number of voxels	p-value at maxima	Coordinates of maxima (x y z)	Location of maxima
		10	44	0.049	-20 -55 54	L Corticospinal tract
		11	24	0.049	-34 -41 43	L Superior Longitudinal Fasciculus
		12	20	0.049	42 -73 -8	R Optic Radiation
		13	17	0.05	-34 -61 32	L Inferior Parietal Cortex
		14	13	0.046	-44 -48 40	L Superior Longitudinal Fasciculus
		15	12	0.029	-13 -71 -36	L Cerebellum
		16	5	0.049	-33 -72 38	L Inferior Parietal Cortex
		17	3	0.05	-34 -63 -35	L Cerebellum
		18	3	0.049	-9 -79 3	L Optic Radiation
		19	2	0.05	-20 -66 -34	L Cerebellum
		20	2	0.05	-37 -64 -34	L Cerebellum
		21	2	0.05	-17 -67 -33	L Cerebellum
		22	1	0.05	-36 -61 -35	L Cerebellum
		23	1	0.05	39 -67 -4	R Optic Radiation
**MD (direct)**	**333088**	**1**	**41636**	**<0.001**	**11 29 -8**	**R Cingulum**
**RD (direct)**	**291888**	**1**	**34836**	**<0.001**	**11 28 -7**	**R Cingulum**
		**2**	**510**	**0.046**	**53 -52 9**	**R Superior Longitudinal Fasciculus**
		3	241	0.047	-46 -58 7	L Superior Longitudinal Fasciculus
		4	239	0.049	39 -22 45	Somatosensory Cortex
		5	206	0.049	44 4 -34	R Inferior Longitudinal Fasciculus
		6	130	0.049	-7 -57 14	L Cingulum
		7	74	0.049	21 9 13	R Anterior Thalamic Radiation
		8	69	0.049	-36 -51 -11	L Inferior Longitudinal Fasciculus
		9	63	0.045	-16 -44 10	Corpus Callosum
		10	60	0.049	-53 -1 -5	L Acoustic Radiation
		11	19	0.05	-53 -42 -5	L Superior Longitudinal Fasciculus
		12	18	0.05	-38 -27 -21	L Inferior Longitudinal Fasciculus
		13	8	0.049	47 23 19	R Superior Longitudinal Fasciculus (frontal)
		14	7	0.05	-7 31 -20	L Cingulum
		15	6	0.05	-31 5 55	L Superior Longitudinal Fasciculus
**MD (inverse)**	**440**	1	38	0.013	-7 -11 -13	L Corticospinal Tract
		2	17	0.001	10 -11 -14	R Corticospinal Tract

Abbreviations: AHEI-2010 - Alternative-Health Eating Index 2010, WHR - waist-to-hip ratio, AD - axial diffusivity, FA - fractional anisotropy, MD - mean diffusivity, RD - radial diffusivity, R - right, L - left.

© 2025 Jensen DEA et al. *JAMA Network Open*

**Cluster sizes (total size in mm^3 and the number of significant voxels), threshold-free cluster enhancement (TFCE) and family-wise error (FWE) corrected p-values, voxel coordinates in MNI-152 space, and locations of cluster maxima are reported for all significant clusters.*

4.2 Results of the ROI analysis of three hippocampal WM fibre tracts

eTable 8: Results of the region of interest analysis showing associations of the fornix, inferior longitudinal fasciculus, and cingulum with AHEI-2010 slope (n=506) and WHR intercept (n=657).

*P-values are markers in bold for p<.05 and with an * for p<.017 for the Bonferroni correction of multiple comparisons across the three white matter tracts.*

	**WM tract**	**WM**	**Beta (SE)**	**CI**	**p-value**
**AHEI-2010 slope**	Fornix	FA	-0.38 (0.19)	-0.75 – -0.02	**0.039**
		MD	0.17 (0.07)	0.03 – 0.30	**0.017**
		RD	0.14 (0.06)	0.02 – 0.25	**0.019**
		AD	0.26 (0.11)	0.05 – 0.47	**0.015***
	ILF	FA	4.32 (2.31)	-0.22 – 8.86	0.062
		MD	-4.03 (1.69)	-7.35 – -0.71	**0.017**
		RD	-3.65 (1.61)	-6.81 – -0.49	**0.024**
		AD	-3.09 (1.48)	-5.99 – -0.19	**0.037**
	Cingulum	FA	0.80 (0.58)	-0.34 – 1.94	0.17
		MD	-1.31 (0.66)	-2.61 – -0.01	**0.049**
		RD	-1.06 (0.56)	-2.16 – 0.04	0.058
		AD	-0.39 (0.43)	-1.24 – 0.45	0.363
**WHR intercept**	Fornix	FA	-0.79 (0.35)	-1.48 – -0.11	**0.024**
		MD	0.18 (0.12)	-0.07 – 0.42	0.156
		RD	0.17 (0.10)	-0.04 – 0.37	0.104
		AD	0.12 (0.19)	-0.26 – 0.49	0.552
	ILF	FA	-14.52 (4.82)	-23.98 – -5.06	**0.003***
		MD	7.21 (3.56)	0.22 – 14.19	**0.043**
		RD	9.11 (3.33)	2.57 – 15.65	**0.006***
		AD	1.28 (3.05)	-4.71 – 7.28	0.674
	Cingulum	FA	-3.08 (1.21)	-5.44 – -0.71	**0.011***
		MD	4.28 (1.34)	1.64 – 6.92	**0.002***
		RD	3.66 (1.15)	1.40 – 5.91	**0.002***
		AD	1.17 (0.86)	-0.52 – 2.87	0.175

Abbreviations: ILF - inferior longitudinal fasciculus, WHR - waist-to-hip ratio, AHEI-2010 - Alternative-Health Eating Index 2010, WM - white matter, AD - axial diffusivity, FA - fractional anisotropy, MD - mean diffusivity, RD - radial diffusivity.

© 2025 Jensen DEA et al. *JAMA Network Open*

5. Hippocampus functional connectivity outcomes

5.1 Cluster report for hippocampal seed-based correlation analysis

eTable 9: Cluster report for seed-based correlation analysis.

*The table shows associations between the intercept and the mean of the AHEI-2010 score (n=512) with functional connectivity of the right and left hippocampus. **

Independent variable	Dependent variable	Cluster size (mm^3)	Cluster number	Number of voxels	p-value at maxima	Coordinates of maxima (x y z)	Location of maxima
**AHEI-2010 (intercept)**	**Right hippocampus connectivity**	**136**	1	17	0.044	40 -80 -30	Right Cerebellum
	**Left hippocampus connectivity**	**9176**	1	331	0.037	-2 -78 -4	Left Occipital Cortex, Lingual Gyrus
			2	310	0.03	-2 -96 -12	Occipital Pole
			3	216	0.035	40 88-16	Lateral Occipital Cortex, inferior division
			4	120	0.037	44 -84 10	Lateral Occipital Cortex, inferior division
			5	111	0.024	20 -90 -18	Occipital Fusiform Gyrus
			6	58	0.042	12 -78 -18	Occipital Fusiform Gyrus
			7	1	0.05	6 -92 28	Occipital Pole
**mean AHEI-2010**	**Left hippocampus connectivity**	**2472**	1	138	0.025	20 -90 -18	Occipital Fusiform Gyrus
			2	71	0.043	12 -78 -18	Occipital Fusiform Gyrus
			3	29	0.049	10 -86 -28	Cerebellum
			4	22	0.047	44 -84 10	Lateral Occipital Cortex, inferior division
			5	17	0.046	-30 -88 -16	Occipital Fusiform Gyrus
			6	12	0.046	-2 -96 -12	Occipital Pole
			7	11	0.05	-36 -78 -40	Cerebellum
			8	7	0.05	-30 -96 -14	Occipital Pole
			9	1	0.05	6 -86 4	Intracalcarine Cortex
			10	1	0.05	8 -84 8	Intracalcarine Cortex

**Cluster sizes (total size in mm3 and the number of significant voxels), threshold-free cluster enhancement (TFCE) and family-wise error (FWE) corrected p-values, voxel coordinates in MNI-152 space, and locations of cluster maxima are reported for all significant clusters.*

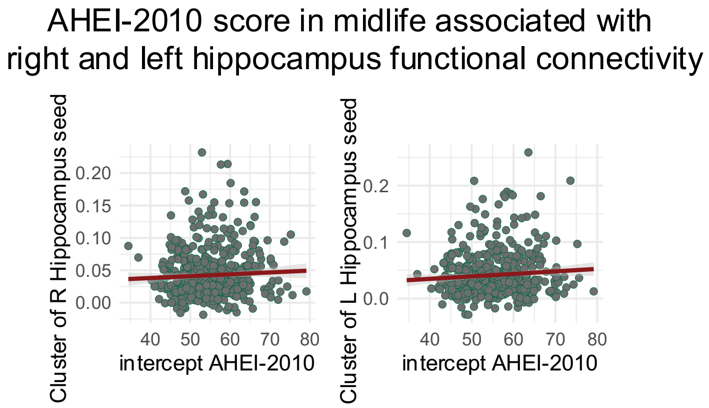

eFigure 7: AHEI-2010 in midlife (i.e. intercept) associated with functional hippocampal connectivity.

Parameter estimates extracted from clusters from right (A) and left (B) hippocampal seed-based correlation analysis outcomes showed positive association with AHEI-2010 score in midlife (i.e. intercept).

© 2025 Jensen DEA et al. *JAMA Network Open*

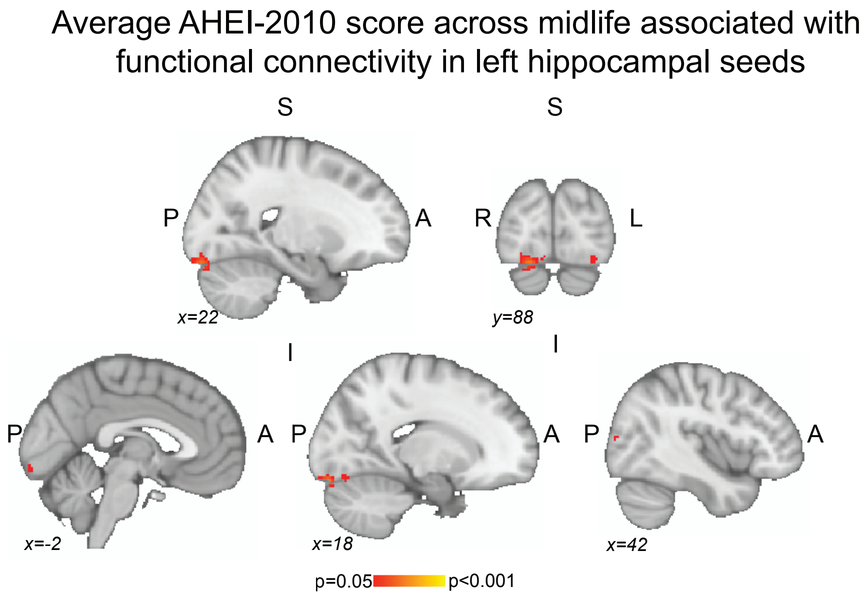

eFigure 8: The association between the average AHEI-2010 score across three waves with left hippocampal functional connectivity in N=512 participants.

Higher functional connectivity of the left hippocampus to clusters in the occipital lobe and the right cerebellum were associated with higher average AHEI-2010 scores. Images show FWE-corrected TFCE statistical maps overlaid on the MNI-152 template. Abbreviations: AHEI-2010 - Alternative-Health Eating Index 2010, A - anterior, P - posterior, S - superior, I - inferior.

6. Association of dietary quality and WHR with cognition

We performed linear regressions between the intercept and slope of AHEI-2010 score and the WHR (eTable 10). No significant associations were found with AHEI-2010 score intercept and slope. However, we found that intercept of WHR was related to working memory (verbal episodic memory (total recall) and digit span test), semantic fluency, and executive function (digit coding and trail making test) performance (see main Fig. 4B).

eTable 10: Associations between the AHEI-2010 score (N=506) and WHR (N=657) and cognitive outcomes.

P-values are markers in bold for p<0.05 and FDR-adjusted p-values (six cognitive outcomes).

	**Cognitive performance**	**β (SE)**	**CI**	**p-value**	**FDR-adjusted p-value**
**intercept AHEI-2010**	Working memory (verbal episodic memory - total recall)	0.01 (0.07)	-0.12, 0.15	0.84	0.88
	Working memory (digit span total)	0.02 (0.06)	-0.09, 0.14	0.7	0.88
	Semantic fluency	0.01 (0.06)	-0.10, 0.13	0.86	0.88
	Lexical fluency	-0.09 (0.07)	-0.23, 0.05	0.22	0.66
	Executive function (digit coding)	0.00 (0.03)	-0.05, 0.05	0.88	0.88
	Executive function (trail making)	-0.95 (0.70)	-2.32, 0.42	0.17	0.66
**slope AHEI-2010**	Working memory (verbal episodic memory - total recall)	0.00 (0.00)	-0.00, 0.01	0.31	0.63
	Working memory (digit span total)	0.00 (0.00)	-0.00, 0.01	0.18	0.55
	Semantic fluency	0.00 (0.00)	-0.01, 0.01	0.92	0.95
	Lexical fluency	0.01 (0.00)	0.00, 0.01	0.03	0.21
	Executive function (digit coding)	0.00 (0.00)	-0.00, 0.00	0.87	0.95
	Executive function (trail making)	-0.00 (0.03)	-0.07, 0.06	0.95	0.95
**intercept WHR**	Working memory (verbal episodic memory - total recall)	-0.02 (0.01)	-0.03, -0.01	0.001	**0.003**
	Working memory (digit span total)	-0.02 (0.01)	-0.03, -0.01	0.001	**0.002**
	Semantic fluency	-0.02 (0.01)	-0.03, -0.01	0.004	**0.007**
	Lexical fluency	0.00 (0.01)	-0.01, 0.01	0.99	0.99
	Executive function (digit coding)	-0.01 (0.00)	-0.01, 0	<0.001	**0.001**
	Executive function (trail making)	0.15 (0.07)	0.01, 0.29	0.03	**0.04**
**slope WHR**	Working memory (verbal episodic memory - total recall)	0.01 (0.01)	-0.00, 0.03	0.16	0.27
	Working memory (digit span total)	0.02 (0.01)	0.00, 0.03	0.03	0.16
	Semantic fluency	-0.01 (0.01)	-0.02, 0.00	0.14	0.27
	Lexical fluency	-0.01 (0.01)	-0.03, 0.01	0.18	0.27
	Executive function (digit coding)	0.00 (0.00)	-0.00, 0.01	0.57	0.69
	Executive function (trail making)	-0.03 (0.09)	-0.20, 0.14	0.74	0.74

Abbreviations: CI - confidence interval.

7. Mediation analyses for WM brain outcomes

In previous analyses we found that the intercept of WHR (but not AHEI-2010 intercept, slope or WHR slope) was related to cognitive outcomes (Y; see eTable 10). Therefore, the mediation analyses was done only for the interaction between WHR intercept and cognitive measures. Here, we tested if that interaction is mediated by white matter integrity using eight mediators (see eTable 11). We found that the association between the intercept of WHR and digit span was mediated by global FA and RD and for digit coding was mediated by global FA, RD and MD (see eFigure 8).
The potential mediation effect of hippocampal function connectivity on the WHR intercept – cognition interaction was not considered as the seed-based correlation analysis did not reveal any potential mediators (see main Results).

eTable 11: Results of 95 % confidence intervals of mediation (1000x Bootstrapping).

*All models are adjusted for confounders (see Methods) and include complete participants' information. Significant mediation effects are indicated in bold, with * for p<0.00625 (Bonferroni correction for multiple comparisons across eight mediators). Tested mediators were: global FA, MD, and RD, ILF (FA, and RD), and Cingulum (FA, MD and RD). We tested the indirect effect when the linear regression of the individual paths (X -> M, and M -> y) showed significant predictions (see Supplementary 1.7)*

Y (cognition)	M (WM brain outcomes)	X -> M (CI)	M -> Y (CI)	Indirect effect (ACME: X -> M -> Y)	Direct effect (ADE: X -> Y)	Total effect (direct + indirect) (X -> Y)	Proportion mediated
**verbal episodic memory**	**global RD**	7751.07 (3642.58, 11859.56), p<0.001	-2.62e-07 (-5.08e-07, -1.53e-08), p=0.04	-2.03e-03 (-4.70e-03, 1.05e-04), p=0.06	-0.02 (-0.03, -7.66e-03), p=0.004	-0.02 (-0.03, -9.61e-03), p=0.002	0.09 (-9.79e-03, 0.27), p=0.06
	**global FA**	-11.72 (-17.49, -5.96), p<0.001	1.84e-04 (8.21e-06, 3.59e-04), p=0.04	-2.15e-03 (-4.87e-03, 5.70e-05), p=0.06	-0.02 (-0.03, -7.65e-03), p=0.002	-0.02 (-0.03, -9.45e-03), p<0.001	0.10 (-3.51e-03, 0.28), p=0.06
	**FA in Cingulum**	-2.84 (-5.20, -0.48), p=0.02	3.64e-04 (-6.90e-05, 7.96e-04), p=0.10	-1.03e-03 (-3.02e-03, 3.29e-04), p=0.16	-0.02 (-0.03, -8.94e-03), p<0.001	-0.02 (-0.03, -9.74e-03), p<0.001	0.05 (-0.02, 0.17), p=0.16
**digit span**	**global MD**	7645.42 (2826.20, 12464.64), p=0.002	-2.76e-07 (-4.47e-07, -1.05e-07), p=0.002	-2.11e-03 (-4.22e-03, -5.00e-04), p=0.01	-0.02 (-0.03, -5.92e-03), p=0.002	-0.02 (-0.03, -8.35e-03), p<0.001	0.11 (0.02, 0.30), p=0.01
	**global RD**	7359.05 (3221.53, 11496.57), p<0.001	-3.65e-07 (-5.64e-07, -1.67e-07), p<0.001	**-2.69e-03 (-5.03e-03, -9.25e-04), p<0.001**	-0.02 (-0.03, -6.36e-03), p=0.002	-0.02 (-0.03, -9.48e-03), p<0.001	**0.14 (0.05, 0.35), p<0.001**
	**global FA**	-11.14 (-16.95, -5.33), p<0.001	2.66e-04 (1.25e-04, 4.07e-04), p<0.001	**-2.96e-03 (-5.56e-03, -1.01e-03), p<0.001**	-0.02 (-0.03, -5.17e-03), p=0.004	-0.02 (-0.03, -8.33e-03), p<0.001	**0.15 (0.05, 0.43), p<0.001**
	**FA in ILF**	-12.49 (-21.97, -3.00), p=0.010	1.49e-04 (6.19e-05, 2.36e-04), p<0.001	-1.86e-03 (-4.14e-03, -3.60e-04), p=0.006	-0.02 (-0.03, -7.44e-03), p=0.006	-0.02 (-0.03, -9.47e-03), p=0.004	0.10 (0.02, 0.26), p=0.01
	**FA in Cingulum**	-2.65 (-5.01, -0.28), p=0.03	4.95e-04 (1.46e-04, 8.45e-04), p=0.006	-1.31e-03 (-3.05e-03, -9.15e-05), p=0.03	-0.02 (-0.03, -6.55e-03), p<0.001	-0.02 (-0.03, -7.72e-03), p<0.001	0.07 (5.14e-03, 0.23), p=0.03

**semantic fluency**	**global RD**	7825.75 (3709.07, 11942.43), p<0.001	-2.21e-07 (-4.26e-07, -1.49e-08), p=0.04	-1.73e-03 (-3.75e-03, -2.20e-04), p=0.03	-0.01 (-0.02, -2.49e-03), p=0.01	-0.02 (-0.03, -4.12e-03), p=0.008	0.11 (0.01, 0.37), p=0.04
	**global FA**	-11.76 (-17.54, -5.98), p<0.001	1.75e-04 (2.88e-05, 3.21e-04), p=0.02	-2.06e-03 (-4.59e-03, -2.49e-04), p=0.02	-0.01 (-0.02, -3.82e-03), p=0.01	-0.02 (-0.03, -5.69e-03), p=0.002	0.13 (0.02, 0.41), p=0.03
	**FA in ILF**	-13.86 (-23.30, -4.43), p=0.004	6.11e-05 (-2.91e-05, 1.51e-04), p=0.18	-8.47e-04 (-2.46e-03, 4.01e-04), p=0.20	-0.01 (-0.03, -4.16e-03), p=0.002	-0.02 (-0.03, -4.93e-03), p=0.002	0.05 (-0.04, 0.19), p=0.20
**digit coding**	**global MD**	7778.52 (2958.04, 12599.00), p=0.002	-1.14e-07 (-1.88e-07, -4.03e-08), p=0.003	**-8.88e-04 (-1.73e-03, -2.40e-04), p=0.004**	-7.65e-03 (-0.01, -3.33e-03), p<0.001	-8.54e-03 (-0.01, -4.06e-03), p<0.001	**0.10 (0.03, 0.24), p=0.004**
	**global RD**	7470.74 (3329.34, 11612.15), p<0.001	-1.55e-07 (-2.41e-07, -6.90e-08), p<0.001	**-1.16e-03 (-2.21e-03, -3.26e-04), p=0.002**	-7.38e-03 (-0.01, -3.17e-03), p=0.004	-8.54e-03 (-0.01, -4.09e-03), p=0.002	**0.14 (0.03, 0.31), p=0.004**
	**global FA**	-11.23 (-17.05, -5.41), p<0.001	1.18e-04 (5.73e-05, 1.79e-04), p<0.001	**-1.33e-03 (-2.38e-03, -5.18e-04), p=0.002**	-7.21e-03 (-0.01, -2.74e-03), p=0.002	-8.54e-03 (-0.01, -4.08e-03), p<0.001	**0.16 (0.06, 0.35), p=0.002**
	**FA in ILF**	-13.01 (-22.47, -3.55), p=0.007	5.24e-05 (1.46e-05, 9.01e-05), p=0.007	-6.82e-04 (-1.46e-03, -9.32e-05), p=0.02	-7.86e-03 (-0.01, -3.07e-03), p=0.002	-8.54e-03 (-0.01, -3.77e-03), p=0.002	0.08 (0.01, 0.22), p=0.03
	**FA in Cingulum**	-2.47 (-4.85, -0.09), p=0.04	3.23e-04 (1.73e-04, 4.74e-04), p<0.001	-7.99e-04 (-1.89e-03, 7.41e-06), p=0.05	-7.74e-03 (-0.01, -3.49e-03), p<0.001	-8.54e-03 (-0.01, -4.45e-03), p<0.001	0.09 (-6.18e-04, 0.26), p=0.05
**trail making**	**global FA**	-11.93 (-17.76, -6.09), p<0.001	-2.07e-03 (-3.87e-03, -2.73e-04), p=0.02	0.02 (1.01e-03, 0.06), p=0.04	0.12 (-0.02, 0.26), p=0.08	0.14 (9.30e-03, 0.27), p=0.04	0.17 (-0.02, 0.75), p=0.07
	**FA in ILF**	-13.78 (-23.32, -4.24), p=0.005	-7.90e-04 (-1.90e-03, 3.17e-04), p=0.16	0.01 (-7.41e-03, 0.03), p=0.23	0.13 (-2.70e-03, 0.27), p=0.06	0.14 (0.01, 0.28), p=0.04	0.08 (-0.11, 0.53), p=0.26

Abbreviations: WM - white matter, FA - fractional anisotropy, MD - mean diffusivity, RD - radial diffusivity, X - predictor variable, y - outcome variable, M - mediator variable, ILF - inferior longitudinal fasciculus, CI - confidence interval, ACME - average causal mediation effect.

© 2025 Jensen DEA et al. *JAMA Network Open*

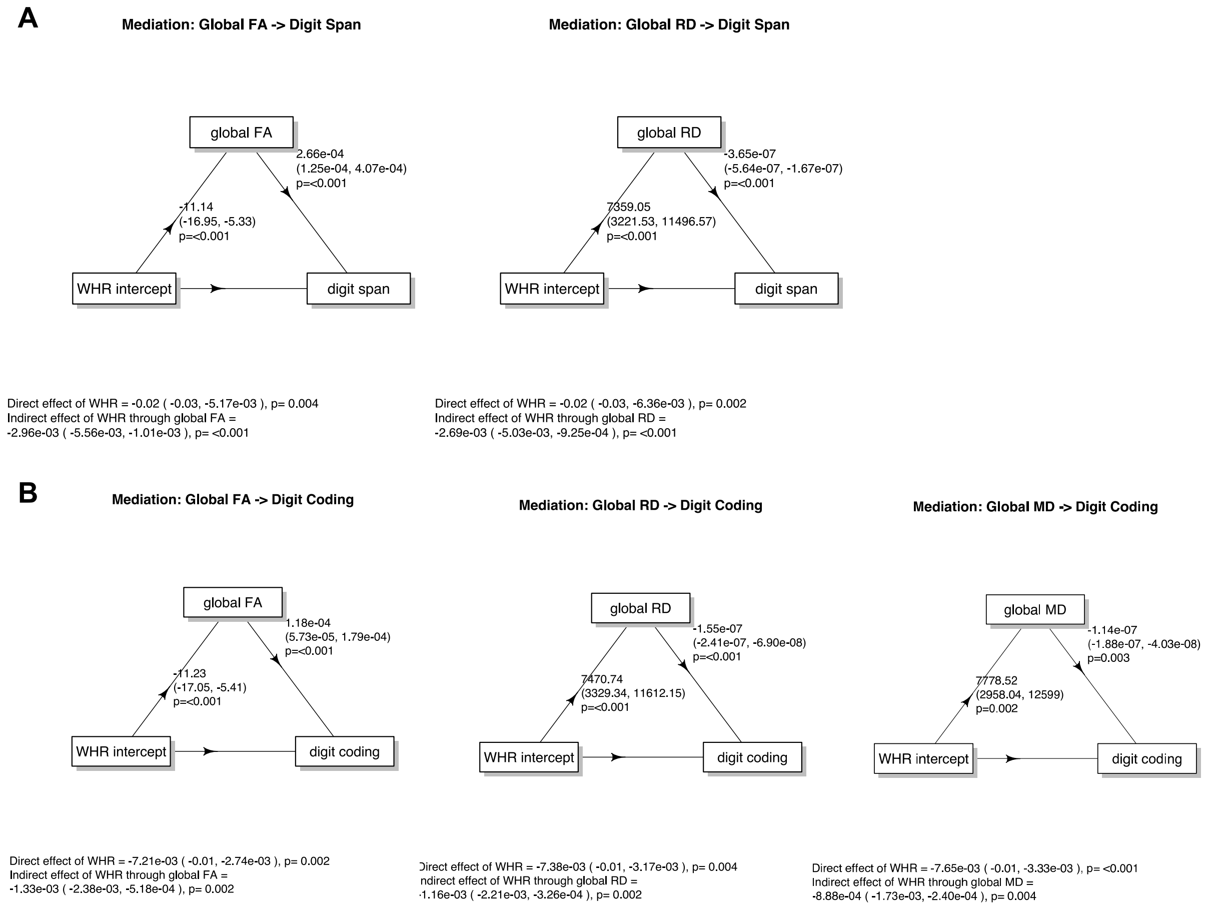

eFigure 9: White matter integrity partially mediates the association between WHR in midlife and digit span and digit coding.

Causal mediation analysis showed that the WHR in midlife (i.e. intercept) has a direct and indirect effect on digit span (A) and digit coding (B), which was partially mediated by global white matter FA (left), RD (middle), and MD (right). Abbreviations: WHR - waist-to-hip ratio, FA - fractional anisotropy, RD - radial diffusivity, MD - mean diffusivity.

© 2025 Jensen DEA et al. *JAMA Network Open*

eReferences

1. Filippini N, Zsoldos E, Haapakoski R, et al (2014) Study protocol: The Whitehall II imaging sub-study. BMC Psychiatry 14:159. https://doi.org/10.1186/1471-244X-14-159

2. Jenkinson M, Beckmann CF, Behrens TEJ, et al (2012) FSL. NeuroImage 62:782–790. https://doi.org/10.1016/j.neuroimage.2011.09.015

3. Smith SM, Jenkinson M, Woolrich MW, et al (2004) Advances in functional and structural MR image analysis and implementation as FSL. NeuroImage 23:S208–S219. https://doi.org/10.1016/j.neuroimage.2004.07.051

4. Zhang Y, Brady M, Smith S (2001) Segmentation of brain MR images through a hidden Markov random field model and the expectation-maximization algorithm. IEEE Transactions on Medical Imaging 20:45–57. https://doi.org/10.1109/42.906424

5. Salimi-Khorshidi G, Douaud G, Beckmann CF, et al (2014) Automatic denoising of functional MRI data: Combining independent component analysis and hierarchical fusion of classifiers. NeuroImage 90:449–468. https://doi.org/10.1016/j.neuroimage.2013.11.046

6. Griffanti L, Salimi-Khorshidi G, Beckmann CF, et al (2014) ICA-based artefact removal and accelerated fMRI acquisition for improved resting state network imaging. NeuroImage 95:232–247. https://doi.org/10.1016/j.neuroimage.2014.03.034

7. Behrens TEJ, Woolrich MW, Jenkinson M, et al (2003) Characterization and propagation of uncertainty in diffusion-weighted MR imaging. Magn Reson Med 50:1077–1088. https://doi.org/10.1002/mrm.10609

8. Akbaraly T, Sexton C, Zsoldos E, et al (2018) Association of long-term diet quality with hippocampal volume: longitudinal cohort study. The American Journal of Medicine. https://doi.org/10.1016/J.AMJMED.2018.07.001

9. Elhakeem A, Hughes RA, Tilling K, et al (2022) Using linear and natural cubic splines, SITAR, and latent trajectory models to characterise nonlinear longitudinal growth trajectories in cohort studies. BMC Medical Research Methodology 22:68. https://doi.org/10.1186/s12874-022-01542-8

10. Greene WH (2003) Econometric Analysis*. Pearson Education. Junger, Washington L., and Antonio Ponce de Leon. 2011. *Ares: Environment Air Pollution Epidemiology: A Library for Timeseries Analysis*.

11. Smith SM, Jenkinson M, Johansen-Berg H, et al (2006) Tract-based spatial statistics: Voxelwise analysis of multi-subject diffusion data. NeuroImage 31:1487–1505. https://doi.org/10.1016/j.neuroimage.2006.02.024

12. Vasconcelos L de G, Brucki SMD, Jackowiski AP, Bueno OFA (2009) Diffusion tensor imaging for Alzheimer's disease: A review of concepts and potential clinical applicability. Dement Neuropsychol 3:268–274. https://doi.org/10.1590/S1980-57642009DN30400002

13. Maller JJ, Welton T, Middione M, et al (2019) Revealing the Hippocampal Connectome through Super-Resolution 1150-Direction Diffusion MRI. Sci Rep 9:2418. https://doi.org/10.1038/s41598-018-37905-9

14. Dale AM, Fischl B, Sereno MI (1999) Cortical Surface-Based Analysis: I. Segmentation and Surface Reconstruction. NeuroImage 9:179–194. https://doi.org/10.1006/nimg.1998.0395

15. Winkler AM, Ridgway GR, Webster MA, et al (2014) Permutation inference for the general linear model. NeuroImage 92:381–397. https://doi.org/10.1016/j.neuroimage.2014.01.060

16. Woolrich MW, Ripley BD, Brady M, Smith SM (2001) Temporal Autocorrelation in Univariate Linear Modeling of FMRI Data. NeuroImage 14:1370–1386. https://doi.org/10.1006/nimg.2001.0931

© 2025 Jensen DEA et al. *JAMA Network Open*

17. Stewart M, Craig D, MacPherson K, Alexander S (2001) Promoting Positive Affect and Diminishing Loneliness of Widowed Seniors Through a Support Intervention. Public Health Nursing 18:54–63. https://doi.org/10.1046/j.1525-1446.2001.00054.x

© 2025 Jensen DEA et al. *JAMA Network Open*
