# Supplementary material for: Association of Diet and Waist-to-Hip Ratio With Brain Connectivity and Memory in Aging
Source: JAMA Netw Open. 2025 Mar 12;8(3):e250171. doi: 10.1001/jamanetworkopen.2025.0171 (PMC11904738; doi:10.1001/jamanetworkopen.2025.0171)
Supplement: Supplement 2. — Data Sharing Statement [file jamanetwopen-e250171-s002.pdf]

Data Sharing Statement

Jensen. Diet and Waist-to-Hip Ratio and Brain Connectivity and Memory in Aging. *JAMA Netw Open*. Published March 12, 2025. doi:10.1001/jamanetworkopen.2025.0171

Data
Data available: Yes
Data types: Other (please specify)
Additional Information: All data that support the findings of this study are available by application to the Whitehall II Study and the Whitehall II Imaging Sub-Study on the DPUK portal (https://portal.dementiasplatform.uk/Apply). As restrictions apply to the availability of these data, which were used under license for the current study, the authors cannot publicly share this data. Uncorrected statistical images from the neuroimaging analyses have been made available at https://identifiers.org/neurovault.collection:19079. Code that allows DPUK users to replicate analyses, including plotting all figures presented in the manuscript is provided in the github repository https://github.com/dariajensen/DietWHRConnectivity. Intermediate analysis outputs can be made available to registered DPUK users upon request and after approval of a proposal. Please see the README file in the Scripts folder for further details.
How to access data: https://portal.dementiasplatform.uk/Apply
When available: With publication

Supporting Documents
Document types: Statistical/analytic code
How to access documents: https://github.com/dariajensen/DietWHRConnectivity
When available: With publication

Additional Information
Who can access the data: All data that support the findings of this study are available by application to the Whitehall II Study and the Whitehall II Imaging Sub-Study on the DPUK portal (https://portal.dementiasplatform.uk/Apply). As restrictions apply to the availability of these data, which were used under license for the current study, the authors cannot publicly share this data. Uncorrected statistical images from the neuroimaging analyses have been made available at https://identifiers.org/neurovault.collection:19079. Code that allows DPUK users to replicate analyses, including plotting all figures presented in the manuscript is provided in the github repository https://github.com/dariajensen/DietWHRConnectivity. Intermediate analysis outputs can be made available to registered DPUK users upon request and after approval of a proposal. Please see the README file in the Scripts folder for further details.
Types of analyses: for any purpose
Mechanisms of data availability: after approval of a proposal
